# Proteomic and Transcriptomic Responses Enable Clams to Correct the pH of Calcifying Fluids and Sustain Biomineralization in Acidified Environments

**DOI:** 10.3390/ijms232416066

**Published:** 2022-12-16

**Authors:** Caroline Schwaner, Sarah Farhat, John Haley, Emmanuelle Pales Espinosa, Bassem Allam

**Affiliations:** 1 School of Marine and Atmospheric Sciences, Stony Brook University, Stony Brook, NY 11794, USA; 2 Biological Mass Spectrometry Center, Stony Brook Medicine, Stony Brook University, Stony Brook, NY 11794, USA

**Keywords:** extrapallial fluid, hemolymph, hemocytes, clams, ocean acidification, biomineralization, transcriptomics, proteomics

## Abstract

Seawater pH and carbonate saturation are predicted to decrease dramatically by the end of the century. This process, designated ocean acidification (OA), threatens economically and ecologically important marine calcifiers, including the northern quahog (*Mercenaria mercenaria*). While many studies have demonstrated the adverse impacts of OA on bivalves, much less is known about mechanisms of resilience and adaptive strategies. Here, we examined clam responses to OA by evaluating cellular (hemocyte activities) and molecular (high-throughput proteomics, RNASeq) changes in hemolymph and extrapallial fluid (EPF—the site of biomineralization located between the mantle and the shell) in *M. mercenaria* continuously exposed to acidified (pH ~7.3; *p*CO_2_ ~2700 ppm) and normal conditions (pH ~8.1; *p*CO_2_ ~600 ppm) for one year. The extracellular pH of EPF and hemolymph (~7.5) was significantly higher than that of the external acidified seawater (~7.3). Under OA conditions, granulocytes (a sub-population of hemocytes important for biomineralization) were able to increase intracellular pH (by 54% in EPF and 79% in hemolymph) and calcium content (by 56% in hemolymph). The increased pH of EPF and hemolymph from clams exposed to high *p*CO_2_ was associated with the overexpression of genes (at both the mRNA and protein levels) related to biomineralization, acid–base balance, and calcium homeostasis, suggesting that clams can use corrective mechanisms to mitigate the negative impact of OA.

## 1. Introduction

Global ocean acidification (OA), caused by anthropogenic CO_2_ uptake, leads to reductions in pH, carbonate concentration, and saturation state (Ω) of calcium carbonate (CaCO_3_) minerals [1,2]. Coastal marine systems face additional disruptions to carbonate system equilibrium (i.e., CO_2_ production from microbial degradation of organic matter and freshwater input), further exacerbating this issue [3]. This is of particular concern to marine calcifying bivalves that need calcium and carbonate ions to build their shells, making them at risk of shell dissolution if seawater pH drops too low [4,5,6]. One such vulnerable marine bivalve is the hard clam or northern quahog, *Mercenaria mercenaria*. The hard clam is one of the most valuable commercial species on the East Coast of the United States, supporting a 60-million-dollar industry [7]. In addition to their economic value, clams (and other suspension-feeding bivalves) provide many ecosystem services, including filtering seawater and prevention of (harmful) algal blooms [8].

Multiple studies have been conducted over the past decade to evaluate the consequences of OA across many taxa [1,4,9,10]. While many of these investigations have shown adverse impacts of OA, there is much variability between species, populations, individuals, and even specific responses. Sometimes neutral effects or even short-term positive effects on particular physiological processes have been demonstrated in response to OA, suggesting that resilience and acclimation mechanisms exist, allowing organisms to overcome OA stress.

What confers resilience to OA is relatively unknown. Most efforts to understand the effects of OA on the hard clam, and bivalves in general, have been focused on the physiological effects of low pH on survival and growth, while overlooking the processes that enable them to survive and tolerate OA. *M. mercenaria* are found in estuarine habitats, such as coastal embayments around Long Island, NY, that currently reach low-pH conditions (pH ~6.3 in extreme cases), so resilience-related mechanisms are extremely relevant [11]. Under normal conditions, bivalves maintain their internal pH below seawater pH [12]. However, it has recently been demonstrated that at low seawater pH some bivalves can increase their pH above that of seawater. Ramesh et al. (2017) found that pH at the calcification site of blue mussel (*Mytilus edulis*) larvae was higher than the seawater pH [13]. The intracellular pH (pH_i_) of hemocytes in OA conditions increased significantly in the Pacific oyster (*Crassostrea gigas*; [14,15]). Similarly, Mediterranean mussels (*Mytilus galloprovincialis*) compensated intracellular acidosis by increasing pH_i_ after 15 days of OA exposure [16]. This ability to regulate pH likely contributes to the mitigation of the negative impacts of OA, allowing bivalves to sustain mineralization despite unfavorable conditions in the external environment. However, this is not universal across bivalve species and there is much variability in this response, with some species unable to modulate calcifying fluids and others showing a limited control that wanes over time or under more severe conditions. For example, the European abalone (*Haliotis tuberculata*) was able to maintain normal extracellular pH (pH_e_) at a seawater pH of 7.7, but this was not sustainable at a pH of 7.4 [17]. The eastern oyster (*Crassostrea virginica*) was able to increase the pH of the extrapallial fluid (EPF—the site of shell formation located between the mantle and the shell) under OA conditions at nine days of exposure, but this ability diminished over time [18]. Some bivalves have not displayed any ability to moderate their internal pH in response to OA. In a study of the blood clam (*Tegillarca granosa*), OA exerted a significant impact on the pH of the hemolymph (invertebrate blood), which decreased as seawater pH decreased [19]. Similarly, Zittier et al. (2015) demonstrated that hemolymph and EPF pH also declined with decreasing pH in *M. edulis* [20]. To further convolute our understanding, even if EPF pH_e_ is maintained, this does not necessarily mean that other fluids respond similarly. For instance, hemolymph pH, but not EPF pH, of *M. galloprovincialis* was significantly impacted by low pH [21]. EPF is the site where biomineralization takes place; however, hemolymph also plays a role in shell formation by mediating the transport of material needed for shell formation [22,23]. The acid–base balance of hemolymph is impacted by OA, which can have adverse effects if not corrected. *M. mercenaria* might be more tolerant to OA if they were able to regulate the pH levels of calcifying fluids, but to date, no studies have investigated this ability in hard clams.

While the ability to correct internal pH in bivalves may confer resilience with respect to OA, it remains unclear whether such compensatory mechanisms come at the cost of other vital functions. A study on the brittle star (*Ophiura ophiura*) showed that there was a trade-off between maintaining calcification under OA and muscle mass [24]. For bivalves, EPF is actually involved in immune response as well as calcification. Hemocytes, which populate EPF and hemolymph, contribute to both biomineralization and immunity [22,23,25]. Therefore, environmental stressors, such as OA, could represent significant challenges for organisms that must integrate biomineralization with maintenance of innate immune protection. Studies have already demonstrated that immunity might be a cost of surviving under OA. For example, the phagocytic activity of hemocytes declined in *M. edulis* under low seawater pH [26,27]. Total hemocyte count (THC) and phagocytic activity of hemocytes was significantly reduced in *T. granosa* under low pH [28]. Pathogen challenges under OA conditions led to increased mortality, suggesting immunosuppression, in *C. gigas* [29] and *M. mercenaria* [30].

At the molecular level, costs of tolerating OA can be seen in the downregulation of specific pathways. For example, sea urchin (*Strongylocentrotus purpuratus*) larvae exposed to OA had decreased expression of a Na^+^/Ca^2+^ exchanger, a Na^+^/K^+^/Ca^2+^ exchanger, and Ca^2+^-ATPase, suggesting alterations in ion regulation and a reduction in the capacity to transport calcium to the sites of calcification [31]. Similarly, sea urchin larvae exposed to OA displayed a downregulation of calcium metabolism proteins (troponin C protein and calmodulin genes) that was associated with slow shell growth and decrease in calcification [32]. Finally, the downregulation of cytoskeletal and signal transduction proteins in larval *Crassostrea hongkongensis* suggests that tolerance to OA comes at the cost of impaired cellular dynamics and organelle development [33]. In parallel, the analysis of transcriptomes/proteomes can also show adaptive mechanisms underlying resistance to OA. For instance, upregulated pathways identified in *Limacina helicina antarctica* (Antarctic pteropod) exposed to OA included biomineralization, calcium ion binding, and DNA binding [34]. Chandra Rajan et al. (2021) found that the overexpression of calcium binding/signaling genes in the mantle was critical to maintaining biomineralization under elevated *p*CO_2_ in the oyster (*C. hongkongensis*) [35].

Despite the growing knowledge of organismal responses to OA, few studies have explicitly examined physiological and molecular responses to OA in body fluids supporting calcification. Here, we examined cellular and molecular responses in the EPF (the fluid where shell formation occurs) and hemolymph (essential for the transport of material to the mineralization site) in adult *M. mercenaria* exposed to OA for one year. The results showed that *M. mercenaria* exposed to OA are able to maintain pH levels of calcifying fluids (pH_e_ of both EPF and hemolymph) well above that of external seawater. In parallel, immune activities (cellular assays) and transcriptomic profiles (RNASeq) of hemocytes from EPF and hemolymph and proteomic profiles (LC/MS-MS on plasma and cell-free EPF) were assessed to better understand how clams regulate their pH under OA and identify any possible physiological trade-offs.

## 2. Results

### 2.1. In Vivo Extracellular pH (pH_e_)

**Figure 1:**
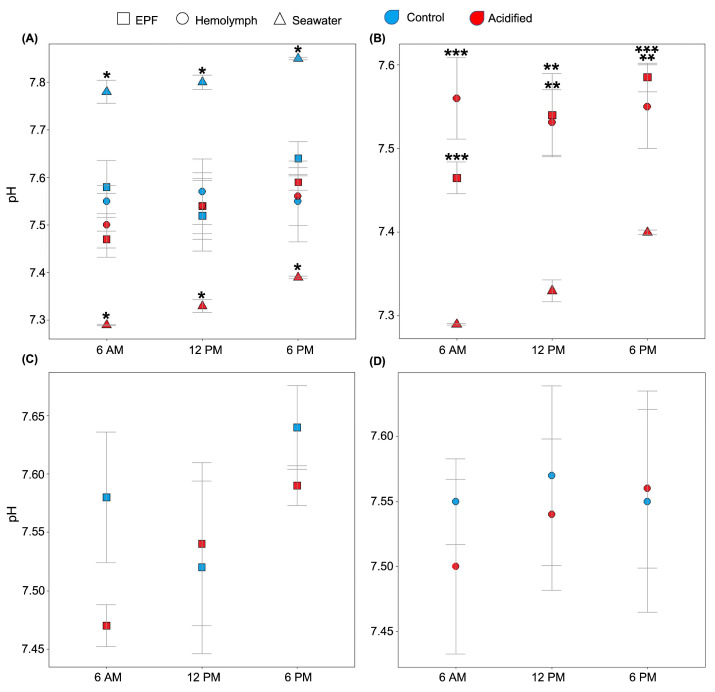
EPF (squares) and hemolymph (circles) pHs of clams under different *p*CO_2_ conditions (red: acidified; blue: control) were measured over a 12 h period. (**A**) Seawater (triangles) pH from both conditions was significantly different from the EPF and hemolymph pHs of the corresponding clams (asterisks denotes significant differences). (**B**) Under the OA conditions, the pHs of both EPF and hemolymph were significantly higher than seawater pH at every time point (* <0.05; ** <0.01; *** <0.001). (**C**) There were no significant differences between EPF pHs of clams in OA and control conditions. (**D**) There were no significant differences between hemolymph pHs of clams in OA and control conditions. n = 4; Student’s *t*-test; *p* < 0.05; means ± standard errors.

We tested the hypothesis that clams can maintain the pH_e_ of EPF and hemolymph despite reductions in seawater pH. The expected diel cycle of the pH in natural seawater was visible, as the daily pH in the control and acidified treatments varied from 7.78 and 7.29 at 6 AM to 7.80 and 7.33 at 12 PM to 7.88 and 7.40 at 6 PM, respectively. Despite these diel variations in seawater pH, EPF and hemolymph systematically maintained different pHs from seawater for both *p*CO_2_ conditions (Figure 1A). The pHs of both EPF and hemolymph from clams maintained in OA conditions were significantly higher than the seawater pH at all measured time points (Figure 1B; *p* < 0.01; Student’s *t*-tests). Overall, EPF pH was more variable and increased in a linear relationship with that of seawater and was almost significantly different between points (*p* = 0.07; Figure 1B). Interestingly, the pHs of the EPF and hemolymph of clams grown under acidified seawater were similar to those measured in clams from the control conditions (Figure 1C,D; *p*-values < 0.05; Student’s *t*-test). While EPF pH tended to increase throughout the day, hemolymph pH remained constant, with no significant differences between time points.

### 2.2. Hemocyte Functional Assays

**Figure 2:**
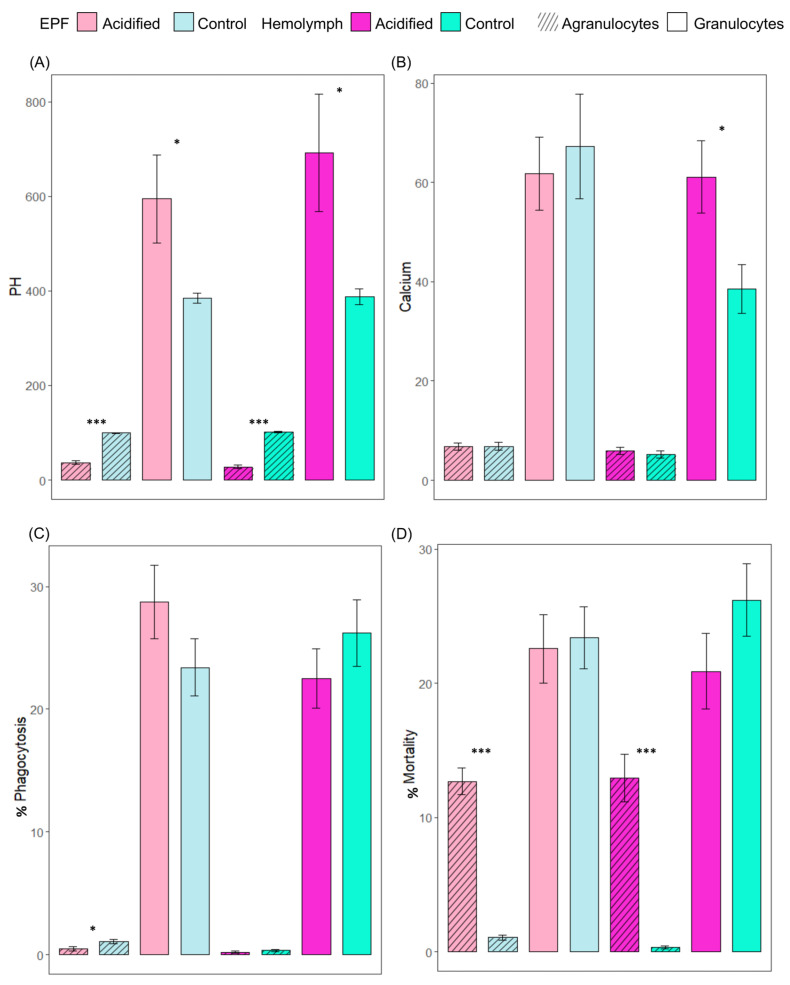
Relative fluorescence intensity of (**A**) BCECF-AM (indicator of intracellular pH) and (**B**) Fluo-3 (indicator of Ca^2+^), and phagocytic activity (**C**) for agranulocytes and granulocytes from EPF and hemolymph. (**D**) Cell mortality (% of PI-positive cells). Shades of pink: acidified; shades of blue: control; lighter shades: EPF; darker shades: hemolymph; diagonal lines: agranulocytes; plain bars: granulocytes. Nested ANOVAs; n = 4; means ± standard errors; asterisks denote significant differences between acidified and control clams (* <0.05; *** <0.001).

We tested the hypothesis that OA exerts significant impacts on hemocyte activities. Like the pH_e_ of EPF and hemolymph, the intracellular pH of hemocytes was measured to see whether pH regulation is sustained as a mechanistic response to low pH of external seawater. Intracellular pHs of granulocytes from EPF and hemolymph from clams maintained in acidified seawater were significantly higher than the pHs of granulocytes from clams maintained in control conditions (*p* = 0.03 and *p* = 0.02, respectively). The reverse trend was found in agranulocytes of EPF and hemolymph, with significantly lower pHs in acidic clams compared to controls (*p* < 0.001) (Figure 2A). Next, the impact of OA on the intracellular calcium contents of hemocytes from EPF and hemolymph was measured. Intracellular calcium of EPF granulocytes and agranulocytes was not significantly different between clams in different *p*CO_2_ conditions. In hemolymph, granulocytes had significantly higher calcium contents under OA conditions (*p* = 0.02), but there were no differences in agranulocytes (*p* = 0.48) (Figure 2B). Lastly, immune parameters of hemocytes in EPF and hemolymph were measured in response to OA to see whether immunity is a cost of tolerance to OA. The percentage of granulocytes (*p* = 0.17) performing phagocytosis in the EPF was not different, but there was a significantly lower percentage of agranulocytes (*p* = 0.03) performing phagocytosis under OA conditions (Figure 2C). The percentages of dead granulocytes from EPF and hemolymph were not significantly different between clams from different treatments. The percentages of dead agranulocytes from EPF and hemolymph were significantly higher than that of granulocytes from clams maintained in control conditions (<0.001) (Figure 2D). There were no differences in the percentages of granulocytes and agranulocytes in EPF or hemolymph between clams from OA and normal conditions (data not shown).

### 2.3. Histopathology and qPCR

Clams were exposed to unfiltered seawater and thus naturally occurring pathogens for one year. Disease and signs of infection in clams were analyzed using standard histopathology procedures. There was no evidence of disease or infection in clams reared in OA or control conditions. While collecting samples for processing, one clam from an OA replicate had a nodule (Figure S1), which is a condition sometimes associated with *Mucochytrium quahogii* infection. The nodule was excised from the clam and used for qPCR diagnostics, but *M. quahogii* was not detected.

### 2.4. RNASeq

Reads mapping to the genome for EPF averaged 14,926,483 ± 1,015,975 and for hemolymph 16,846,207 ± 2,703,850 per clam.

#### 2.4.1. Differential Expression Analysis 

**Figure 3:**
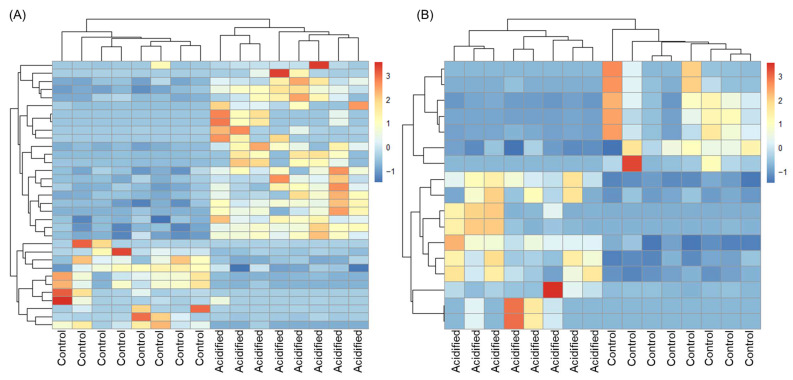
Hierarchical clustering analysis of differentially expressed genes. Over- and under-expressed genes are represented in red and blue, respectively. (**A**) Extrapallial fluid. (**B**) Hemolymph.

We tested the hypothesis that the transcriptome of EPF and hemolymph in clams is sensitive to OA by looking at the response of individual genes via differential gene expression analysis. EPF from clams maintained under high *p*CO_2_ was compared to EPF from clams maintained under control conditions, resulting in 33 genes with log2foldchanges > |2| and adjusted *p*-values < 0.05. Of the differentially expressed genes (DEGs), 20 were upregulated under OA and 13 were downregulated (Table 1 and Table S1). Using hierarchical clustering analysis (Figure 3), EPF samples clustered based on *p*CO_2_ treatment (i.e., EPF samples from clams maintained at high *p*CO_2_ clustered most closely as opposed to EPF samples from clams maintained at control *p*CO_2_). Hemolymph from clams maintained under high *p*CO_2_ was compared to hemolymph from clams maintained under control conditions, resulting in 17 genes with log2foldchanges > |2| and adjusted *p*-values < 0.05. Of the DEGs, 10 were upregulated under OA and 7 were downregulated (Table 1 and Table S2). Similar to EPF, hemolymph samples clustered by *p*CO_2._ In each comparison (EPF OA vs. EPF control and hemolymph OA vs. hemolymph control), five genes overlapped among upregulated DEGs and four among downregulated DEGs (Table 1). OA-induced changes in GO categories were also examined using GO enrichment analysis; however, no GO categories were found to be enriched in DEGs in any comparison.

#### 2.4.2. WGCNA

**Figure 4:**
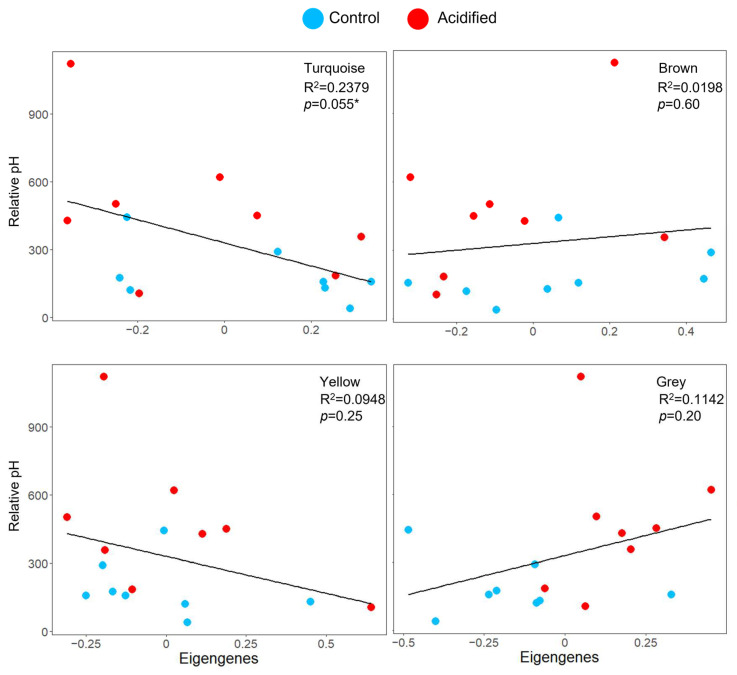
Co-expression responses to OA. Top four modules associated with clam EPF (colors are randomly assigned by the WGCNA package and have no meaning). Scatterplots of the pH_i_ (y-axis, represented as relative fluorescence units measured by the flow cytometer) of EPF hemocytes by eigengene expression (x-axis). R^2^ and *p*-values for the linear regressions are provided. * denotes significance.

Gene co-expression network analysis of EPF identified six modules of co-expressed genes (genes with similar expression patterns among all the clams) ranging from 83 to 577 genes in size (Table S3). These modules were used to test the hypothesis that the pH of EPF is associated with the expression of gene modules. Among these, the “turquoise” module was almost significantly associated with pH_i_ (Figure 4; Table S3; linear regression *p*-value = 0.055). This module was composed of genes related to biomineralization, ion transport, and acid–base regulation. For hemolymph, five modules were identified, ranging from 297 to 533 genes (Table S4). These modules were used to test the hypothesis that the pHs of hemolymph and intracellular calcium were associated with expression modules; however, none were significantly associated. 

Using GO enrichment analysis, it was tested whether OA induced changes in particular GO categories; however, there were no enriched GO terms for any of the clusters.

### 2.5. Proteomics

We tested the hypothesis that the proteome of EPF and hemolymph in clams is sensitive to OA by looking at the response of individual proteins via differential protein expression analysis. EPF from clams maintained under high-*p*CO_2_ was compared with EPF from clams maintained under control conditions, resulting in 125 proteins with log2foldchanges > |0.8|, >1 peptides, and *p*-values < 0.05. Sixty-two proteins were upregulated under OA conditions and 63 were downregulated (Table 2 and Table S5). Hemolymph from clams maintained under high *p*CO_2_ was also compared to hemolymph from clams maintained under control conditions, resulting in 108 proteins with log2foldchanges > |0.8|, >1 peptides, and *p*-values < 0.05, with 49 proteins upregulated under OA conditions and 59 downregulated (Table 2 and Table S6). In each comparison (EPF OA vs. EPF control and hemolymph OA vs. hemolymph control), 13 proteins were upregulated under OA in both fluids, while 19 of the same proteins were downregulated in both fluids (Table 2). OA-induced changes in GO categories were also examined using GO enrichment analysis; however, no GO categories were found to be enriched in DEGs in any comparison. The transcriptome and proteome were not correlated for EPF (regression analysis; *p* = 0.9672), while the correlation was nearly significant for hemolymph (*p* = 0.055).

## 3. Discussion

This study investigated the physiological and molecular responses of calcifying fluids to OA in adult *M. mercenaria*. Clams were able to elevate their EPF (Δ 0.18) and hemolymph pHs (Δ 0.29) at low levels of seawater pH (7.29), which suggests that this might be a mechanistic response to maintain the microenvironment conducive to shell mineralization. OA exerted a significant influence on the pH_i_ of hemocytes in both EPF and hemolymph and on calcium concentration in hemolymph granulocytes. While there were no differences in the immune parameters measured in granulocytes, phagocytosis and viability of agranulocytes were impacted by OA. Investigations of the transcriptomes of hemocytes and the proteomes of cell-free EPF and serum showed shifts in gene expression. Clustering analysis revealed a subtle association between gene expression and pH. Below, we discuss physiological and molecular responses in EPF and hemolymph and how they are related and probe the molecular underpinnings of resilience to OA.

### 3.1. Clams Were Able to Maintain pH_e_ Despite the pCO_2_ of the Seawater 

In this study, both EPF and hemolymph had significantly different pHs from seawater for both *p*CO_2_ conditions. In the OA treatment, the pH_e_s of EPF and hemolymph were significantly higher than the pH of the seawater at all time points, and in control conditions pH_e_s were significantly lower. In his seminal work on EPF, Crenshaw (1972) found that, under normal *p*CO_2_, pH_EPF_ was lower than that of seawater for all bivalve species investigated [12]. This was true for the control clams in our study. pH_e_ seems to remain at optimal pH despite variations in external seawater pH. Patterns of pH changes were similar in hemolymph and EPF, confirming that carbonate saturation status is similar in both body fluids [21,36]. To note, while overall trends were similar for both fluids in this study, EPF did vary more than hemolymph, and EPF had more of a linear relationship within OA conditions, whereas hemolymph remained more constant over the three sampling time points. This indicates that hemolymph is less influenced by external pH. This might be due to the fact that EPF is separated from seawater only by a thin layer of mantle tissue, making it more prone than hemolymph to changes due to the influence of the external ionic environment. Many bivalve species are found in coastal environments that experience altered carbonate chemistry and have some capacity to deal with these changes. However, pH below and *p*CO_2_ above natural minima and maxima could limit their ability to compensate for changes in calcifying fluid chemistry, as has been demonstrated in previous studies [17,19,20,37]. Clams in this experiment were exposed to a dramatic reduction in pH and were maintained for one year, and they were still able to control pH_e_. Bivalves can use buffering and active ion transport to control their acid–base chemistry. They have mechanisms to tolerate alterations in carbonate chemistry, such as the ability to concentrate HCO_3_^−^ in the calcifying fluid through exchange of HCO_3_^−^/Cl^−^ and maintain pH homeostasis through active removal of protons [38]. Non-bicarbonate buffering by histidine residues of plasma proteins [39] can capture some protons and lead to increase in extracellular bicarbonate when seawater pH is low; however, this is dependent on protein concentration. This can help to mitigate the drop in extracellular pH when bivalves are exposed to OA conditions. The other process is accumulation of bicarbonate via ion transport proteins, which can lead to full or partial compensation for OA conditions [40]. Zhao et al. (2018) demonstrated that the soft-shell clam (*Mya arenaria*) maintained pH homeostasis through the proton pump [41]. Analysis of the transcriptome and proteome revealed many genes related to acid–base regulation and ion transport, such as bicarbonate transporters and carbonic anhydrases (discussed below). We hypothesize that the maintenance of pH_e_ may be the outcome of molecular regulation, as shown by marked changes in gene expression (at both mRNA and protein levels), as discussed below.

### 3.2. Intracellular pH and Calcium Varied with Differing pCO_2_

The intracellular pHs of granulocytes from EPF and hemolymph from clams maintained in acidified seawater were significantly higher than the pHs of granulocytes from clams maintained in control conditions; however, the reverse trend was found in agranulocytes. Studies focusing on intracellular pH found compensated or only slightly reduced intracellular pH in marine invertebrates under OA conditions due to higher intracellular buffering capacity and active net bicarbonate accumulation. Marine invertebrates can fully restore intracellular pH during acclimation to OA via bicarbonate accumulation (reviewed in [40]). CO_2_-related decrease in pH_i_ can be fully compensated after 24–48 h in most marine invertebrates and fish [42]. For example, the pH_i_s of hemocytes of *C. gigas* [14,15] and *M. galloprovincialis* [16] in OA conditions increased significantly. The reverse trend in agranulocytes might mean that either they do not have the capacity to compensate for decreases in environmental pH or that it is not necessary for them to do so. Granulocytes are the hemocyte type most closely associated with mineralization [22]. A study examining gene expression of the different sub-populations of hemocytes found differences in the expression of ion transporters between cell fractions [43]. Agranulocytes might lack the complexity and the proteins necessary to respond. The turquoise module was almost significantly correlated with pH_i_, suggesting that this is at least partially controlled by molecular mechanisms (discussed below).

In hemolymph, granulocytes had significantly higher calcium contents under OA conditions, but there were no differences in any other comparison. Granulocytes in hemolymph are associated with calcium transport [22,30]; specifically, hemocytes are able to sequester Ca^2+^ and CO_3_^2−^ and transport them intracellularly to sites of shell formation [22]. Thus, the calcium content of hemocytes is important for CaCO_3_ mineralization and could be a mechanism for repairing shell damage or promoting mineralization under OA conditions. Wang et al. (2017) found the opposite trend to the one observed in this study, with hemocytes from *C. gigas* maintained in elevated *p*CO_2_ conditions having decreased calcium contents [15]. Interestingly, Wang et al. (2020) demonstrated a reduction in intracellular calcium in the short term but showed that the levels recovered to normal values after long-term exposure, suggesting that a mechanism was in place to compensate for the changes in intracellular calcium [44]. Both studies examined hemolymph hemocytes only. The lack of differences in the other comparisons could suggest that the clams have a mechanism to regulate intracellular calcium. In fact, many calcium-binding and -transporting genes were identified and are discussed below. In a comparison of *C. virginica* and *C. gigas*, *C. virginica* hemocytes had greater intracellular calcium as well as upregulation of biomineralization-related genes [45]. The authors suggested that this contributed to better shell quality in *C. virginica*, and a similar regulatory process may help clams protect themselves from OA stress in our study.

### 3.3. Effect of OA on Cellular Immune Functions

The effect of OA on the immune parameters of hemocytes in EPF and hemolymph was evaluated to investigate potential physiological trade-offs of maintaining pH in EPF and hemolymph. The only differences in phagocytosis detected were in EPF agranulocytes, which had a reduced percentage of cells performing phagocytosis under OA conditions. For both EPF and hemolymph, the percentage of dead agranulocytes was significantly higher in clams exposed to OA as compared to that of agranulocytes from control clams. No differences were detected in the percentages of granulocytes and agranulocytes in EPF or hemolymph. While previous studies evaluated hemocyte parameters in hemolymph from bivalves exposed to OA, the evaluation of how OA affects immune parameters in EPF is novel.

Phagocytosis of hemocytes was investigated as it is a major immune response and previous studies have shown a reduction in the phagocytic activity of circulatory hemocytes under OA. For example, phagocytosis was inhibited under elevated *p*CO_2_ in *M. edulis* [26,27] and *T. granosa* [46]. Another study revealed that phagocytosis and total hemocyte counts (THC) were reduced in *T. granosa* upon exposure to elevated *p*CO_2_ [28]. THC was investigated because it can reflect inflammatory mobilization, hemocyte proliferation, and hemocyte recruitment, which is the first cellular response of the immune system [47]. In their study in *M. edulis*, Bibby et al. (2008) showed no change in THC under OA conditions [26]. In contrast, THC was significantly reduced under low-pH conditions in the thick shell mussel (*Mytilus coruscus*; [48]). Potentially, dual stressors of OA and pathogen challenge might induce a more intense response. For instance, larval and juvenile *M. mercenaria* exposed to elevated *p*CO_2_ were susceptible to pathogenic *Vibrio* spp. and had higher mortality than controls [30]. Similarly, *C. gigas* exposed to *Vibrio* spp. and OA showed a decrease in phagocytosis and an increase in the percentage of dead hemocytes [29]. Cao et al. (2018) suggested that the greater percentage of dead hemocytes in *C. gigas* might be due to excessive ROS production under OA stress [28]. In our study, there was a significant reduction in the phagocytic activity of agranulocytes in EPF and a reduction in cell viability for agranulocytes in both EPF and hemolymph, suggesting that there were immune costs to clam maintenance under OA. As hemocytes play dual roles in biomineralization and immunity [22,23], maintaining mineralization might come at a cost to immune function. While EPF is mostly associated with biomineralization, hemocytes in EPF are important components of the immune response [23,49,50]. Under OA conditions, EPF may pivot mostly towards biomineralization processes, which is supported by transcriptomic and proteomic analyses showing upregulation of biomineralization genes and downregulation of immune genes in EPF from clams exposed to acidification (see below). This did not translate, however, into overt infections, as there were no detectable differences in disease prevalence or signs of infection between clams from different *p*CO_2_ conditions. Indeed, bivalve immunity (as for immunity in virtually all metazoan organisms) is multilayered and redundancy exists [51], ensuring that animal health is maintained even if some immune pathways appear depressed.

### 3.4. Gene Expression Revealed Upregulated and Downregulated Pathways

Gene expression in EPF and hemolymph from clams maintained under high *p*CO_2_ was compared to that of clams maintained under control conditions, resulting in the identification of DEGs. After hierarchical clustering analysis, EPF and hemolymph samples clustered based on *p*CO_2_ treatment. EPF had a higher number of DEGs. Since the extrapallial space is the site of shell formation, the chemistry of the calcifying fluid in this compartment is directly impacted by OA. The most biological control in this area is needed to actively modulate the extracellular carbonate system and stabilize pH_e_, whereas hemolymph plays a more peripheral role in biomineralization and so might be less impacted. Despite the differences between these two fluids, in each comparison, there was an overlap of genes upregulated and downregulated under OA conditions. The upregulated genes included carboxylesterases type-B signature 2, two myosin-3-like genes, and two theromacins; the downregulated genes included two integrase catalytic domain profile and two protocadherin Fat 1/2/3 genes.

There is little information on the different types of biomineralizing cells, especially differences in hemocytes between EPF and hemolymph and their specialization in the biomineralization processes. Even less is known about the impacts of OA on these cellular pathways. Here, we tried to use information derived from DEG analysis to elucidate the role of hemocytes in biomineralization and their response to elevated *p*CO_2_ in seawater. Theromacins were upregulated in hemocytes from both EPF and hemolymph from clams under acidified conditions as compared to clams in normal seawater. While theromacins have been associated with immune processes, they have also been shown to play a role in pearl sac formation [52] and were classified as biomineralization-associated genes in the pearl oyster [53]. Theromacins are a family of antimicrobial peptides that have cationic and hydrophobic properties that are synonymous with characteristics of the periostracum and insoluble shell matrix [54,55,56]. It is possible that the poly-anionic glycoproteins (shell precursors) bind to cationic peptides in the periostracum, which helps to activate nucleation sites by which microstructure mineralization occurs [53]. There are several genes that appear to function in both biomineralization and immunity and differentiating between the two can be difficult. 

Myosin-3-like was upregulated in hemocytes from EPF and hemolymph as well. Upregulation of myosin was also found in *C. hongkongensis* larvae [33], *C. gigas* [57], and *M. coruscus* [58] exposed to OA conditions. Upregulated cytoskeleton genes could be a response to the damage to the hemocyte cytoskeleton incurred due to OA [45]. Carrol et al. (2021) demonstrated functional enrichment of cytoskeleton-related genes under OA and suggested that this might be related to cytoskeletal reorganization and stabilization under cellular stress to regain cellular structural integrity [59]. While the role of carboxylesterases in OA response is not so clear, they were found in the turquoise module where genes clustered based on co-expression. The other genes in this group were related to biomineralization, ion transport, and acid–base regulation. 

Genes coding for the protein Cytochrome P450 were upregulated in EPF and hemolymph under OA conditions. Cytochrome P450 was upregulated in CO_2_-resilient Sydney rock oysters (*Saccostrea glomerata*; [60]) and upregulated in *C. gigas* exposed to OA [57]. The upregulation of this gene could occur to cope with oxidative stress induced by elevated cellular CO_2_ and H^+^, since cytochrome P450 has a role in cell protection against oxidative stress.

Solute carrier family 25 and 6 were upregulated under OA in the EPF. The solute carrier family is a group of ion transport proteins that are known to transport HCO_3_^−^ [61]. Their role in bicarbonate transport could be critical to maintaining acid–base balance and might be responsible for maintaining intracellular pH, which was observed in this study. Eleven solute carriers were identified in the turquoise module, which had a correlation with intracellular pH. These genes might be at least partially responsible for regulating intracellular pH through ion and bicarbonate transport.

Downregulated genes in common between EPF and hemolymph included protocadherins. Protocadherins are cell adhesion molecules, but they have been shown to be related to calcium signaling and biomineralization and upregulated in other studies of animals exposed to OA [35]. The downregulation of protocadherin may indicate alterations in calcium homeostasis under OA, and, in fact, intracellular calcium tended to be lower in granulocytes from EPF collected from clams under OA as compared to controls. This could possibly be due to a disruption in calcium signaling via downregulation of protocadherins.

A gene with tumor necrosis factor domain (TNF), an immune gene, was downregulated in EPF. It was also downregulated in wild oysters under OA [60]. This might be indicative of a fitness cost, i.e., immune suppression, or demonstrate a trade-off between biomineralization and immune processes in the EPF. The tubulin alpha gene was also downregulated in EPF. Downregulation of a cytoskeletal gene could explain why there was a decrease in phagocytosis, as this activity is mediated by the cytoskeleton. Su et al. (2018) suggested that the reduction in the phagocytic activity of hemocytes from *T. granosa* under OA may be related to a decrease in the abundance of cytoskeletal proteins [45].

### 3.5. Clustering Analysis Associated with Change in pH_i_

The turquoise module was correlated with the pH_i_ of EPF hemocytes, which increased in response to reduced pH in the external seawater. The genes in this module included all the domains referenced in the “basic toolkit for calcification” [62]: carbonic anhydrase; chitin-binding type 2 domain (also included chitinase and chitin synthase); Von Willebrand Factor A domain (also included the VWFC domain signature); and tyrosinase. The most important of these in relation to pH_i_ would be carbonic anhydrase (CA). Five CAs were among the characterized genes in the turquoise module. CAs catalyze the reversible reaction of carbonate hydration: CO_2_ + H_2_O <--> HCO_3_^−^ + H^+^, and they are important for acid–base regulation and ion transport [63,64]. Under OA conditions, CA can transport H^+^ and HCO_3_^−^ across cell membranes, which would lead to an increase in intracellular pH [15]. Bicarbonate transport proteins (HCO_3_^−^ transporter family) were also found in the turquoise module and would function similarly to CA to increase intracellular pH. In addition, there were many other ion transporters among the characterized turquoise genes. These included 11 solute carriers, a gene belonging to the solute: the sodium symporter (SSS) family, the sodium/hydrogen exchanger family, and sulfate anion transporters. There were also several genes responsible for calcium ion binding and transport. These included five genes with EF-hand calcium-binding domains, six sarcoplasmic-calcium-binding proteins, cadherin, and calmodulin. Upregulating calcium-binding or transport proteins is a well-known mechanism to sustain biomineralization under OA [35,65,66]. There were also several genes involved in calcium carbonate crystal nucleation [62], including a gene with a Whey Acidic Protein (WAP) domain, perlucin-like, and 11 genes with epidermal growth factor (EGF) domains. Other shell formation proteins [62] included genes with metalloproteases, genes with kazal domains, insoluble shell matrix proteins, collagens, and laminin genes. We hypothesize that the uncharacterized genes in this cluster are probably associated with acid–base regulation, ion transport, and biomineralization.

### 3.6. Protein Expression Revealed Upregulated and Downregulated Pathways

OA did appear to significantly influence the proteome of both cell-free EPF and hemolymph plasma. Both fluids showed an upregulation of genes related to calcium binding or transport. As mentioned previously, the upregulation of calcium-related genes is a recognized mechanism for maintaining biomineralization under OA. Sarcoplasmic-calcium-binding protein, a part of the calcium regulatory toolkit [43], was upregulated in both EPF and hemolymph. Sarcoplasmic-calcium-binding protein might be one of the genes underlying calcium homeostasis in hemocytes in clams exposed to OA. This gene was also upregulated in *C. gigas* under long-term CO_2_ exposure [43]. The upregulation of this gene (at the protein level) might explain why we saw similar calcium contents between *p*CO_2_ conditions in EPF agranulocytes, granulocytes, and hemolymph agranulocytes. Sodium/calcium exchanger regulatory protein 1-like was upregulated in EPF and downregulated in hemolymph. This protein is important for ion transport and acid–base regulation. It was identified as being important for the regulation of pH_i_ in *C. gigas* [64]. It might be at least partially responsible for the regulation of intracellular pH observed in this experiment. The same gene with the chitin-binding type-2 domain was upregulated in both EPF and hemolymph. As mentioned before, this is one of the domains identified as part of the basic toolkit for biomineralization [62]. It was also upregulated in larval *C. gigas* during shell formation when exposed to OA [67]. Laminin alpha 3/5, was upregulated in EPF but downregulated in the hemolymph proteome. Laminin alpha 3/5 is a biomineralization gene that has calcium carbonate-binding activity [68] and was found to be upregulated under OA in *C. hongkongensis* [35]. While this biomineralization gene and the sodium/calcium exchanger protein were upregulated in EPF and downregulated in hemolymph, there were genes that displayed the reverse trend, including the immune gene ficolin-1-like and a gene with the fibrinogen C-terminal domain. This might indicate that, under OA conditions, it is more important to maintain pH and biomineralization in the EPF, even if this comes at a cost to immune activities in that fluid. Other immune genes were, however, upregulated in the proteomes of both EPF and hemolymph. One of these, the mannose C-type lectin gene, has been shown to be upregulated in the corals *Desmophyllum dianthus* [69] and *Malacobelemnon daytoni* [70] under elevated *p*CO_2_. This might indicate that OA activates the innate immune system or front-loads the defense repertoire to enhance survival under adverse environmental conditions. A C-type lectin domain is also found in the gene perlucin, which is responsible for CaCO_3_ nucleation. In fact, all C-type lectins rely on calcium ions for activation and may have dual functions in immunity and biomineralization, making it difficult to pinpoint the specific pathway at play here.

In this study, hemocytes and supernatants from EPF and hemolymph were treated differently, i.e., RNASeq was performed on hemocytes and proteomic analysis on cell-free fluids. Proteomic analysis of cell lysate would be less informative, since structural proteins would overwhelm signals generated by genes of interest despite likely being less abundant (dynamic range is better for RNASeq methods as compared to proteomics methods). Further, it is known that a large fraction of functional proteins present in plasma are secreted by hemocytes [50]. In fact, previous work on bivalves has shown that the plasma proteome reflects some of the proteomic make-up of circulatory hemocytes [23,71]. We had hypothesized that there would be a correlation between the two; however, this was not true for EPF and only a slightly significant correlation was found for hemolymph. The lack of correlation in EPF suggests that some, if not many, proteins present in cell-free EPF actually derive from the mantle and not from hemocytes, in agreement with previous suggestions [72].

### 3.7. Lack of Enrichment of Functional Groups 

Gene Ontology (GO) enrichment analysis was used to evaluate whether OA induces changes in particular GO categories, but no such enrichments were identified either by RNASeq or proteomics. It should be noted that GO categories are not optimized for studies on non-model marine invertebrates and that the database is largely built using model organisms that do not possess the traits of interest for this study. In fact, the reliance on GO categories has been called into question by Melzner et al. (2022), and the authors specifically mention that using GO databases is not appropriate for studies of calcifying organisms under climate change stress [73]. In addition, not all of the *M. mercenaria* genes had associated GO terms, which would have skewed the results and might explain why there were no significant differences. 

## 4. Materials and Methods

### 4.1. Animals 

Two hundred adult clams (50–70 mm) were obtained from a commercial source (Frank M. Flowers & Sons Inc., Oyster Bay, NY, USA). The clams were washed and placed in ambient conditions upon their arrival (salinity 30 practical salinity units (PSU), temperature 25 °C, pH 7.8). For their first week they were fed daily a commercial diet (LPB Frozen Shellfish Diet, Reed Mariculture, Campbell, CA, USA). After one week of acclimation, the clams were moved into a flow-through system (described below). They were separated into eight tanks, four tanks per *p*CO_2_ treatment, with 25 clams in each replicate. Once in the flow-through system, the clams only received the algae in the raw water without supplemental feeding. At 10 months—a time chosen based on dramatic diel changes in pH—a subsample of clams was sampled to monitor extracellular pH over a period of 12 h. At the end of one year of exposure to acidified or ambient (control) treatments, the clams were sampled for the remaining assays.

### 4.2. Seawater Chemistry 

The clams were held in an open flow-through system as described in Schwaner et al. (2020) [30]. In coastal estuarine habitats, environmental parameters fluctuate on both diel and seasonal scales. Ignoring natural fluctuations can weaken the relevance of findings. The flow-through design incorporated both diel and seasonal fluctuations rather than just comparing stable mean pH levels. For the ambient condition, the average pH over the year was ~8.01, with a corresponding *p*CO_2_ of ~600 ppm, although the pH dropped lower in summer and early fall. For the OA condition, the average pH was ~7.27, with a corresponding *p*CO_2_ of ~2700 ppm. The chemistry within the acidified treatment was more extreme than predictions for the open ocean at the end of the century [74] because ambient conditions in eutrophic estuaries surpass these predictions in summer months [11]. For instance, pH recorded in a eutrophic estuary on Long Island ranged from maxima of 8.28–8.06 to minima of 7.87–7.15 [11]. To maintain high-*p*CO_2_/low-pH conditions for the OA treatment, seawater flowed into an acidification chamber [30], where 100% CO_2_ gas was mixed with air using multichannel gas proportioners (Cole Parmer^®^ Flowmeter system, multitube frame; Antylia Scientific; Vernon Hills, IL, USA). CO_2_ gas and air were bubbled into the chamber continuously to maintain a delta of ~0.7 pH units (the predicted decrease in pH under the “business-as-usual” scenarios [75]); between the two treatments. After equilibrating to the desired condition, water from the chamber flowed into four replicate vessels corresponding to the high-*p*CO_2_/low-pH treatment using a “downweller” setting. To reflect natural conditions, water flowed directly from the source (Old Fort Pond, Southampton, NY, USA) into an aerated head tank and then continuously flowed into each of four replicate tanks for the ambient treatment. Each of the replicate tanks per *p*CO_2_ treatment was an experimental unit (n = 4). *p*CO_2_ treatment was the fixed effect and the replicate tank was the random effect, following recommendations in Cornwall and Hurd (2016) [76]. Tanks holding the clams and tubing were regularly cleaned (approximately 3 to 5 times a week) to prevent biofouling and waste collection and to maintain constant flow (acidification chamber cleaned regularly; frequency depending on season). The pH was monitored daily using a Durafet III pH probe (Honeywell, Morristown, NJ, USA). Seawater samples for dissolved inorganic carbon (DIC) analysis were collected and read using a VINDTA 3D (Versatile Instrument for the Determination of Total Inorganic Carbon; manufactured by Ludger Mintrop, Marianda, Kiel, Germany) delivery system coupled to a UIC Inc. (Joliet, IL, USA) coulometer (model CM5017O). Bicarbonate standards were used, and certified reference material was analyzed (provided by Andrew Dickson, Scripps Institution of Oceanography; La Jolla, CA, USA) with a 99.99% recovery during every run for quality assurance. *p*CO_2_, Ω_aragonite_, Ω_calcite_, DIC, CO_3_, and alkalinity were calculated from pH, temperature, and salinity using the *seacarb* package [77] for R statistical software v.4.2.0 (R core team, Vienna, Austria), following parameters recommended by Dickson et al. (2007) [78] with known first and second dissociation constants of carbonic acid in seawater [79]. Seawater chemistry characteristics are provided in Table S7.

### 4.3. In Vivo Measurement of Extracellular pH (pH_e_)

**Figure 5:**
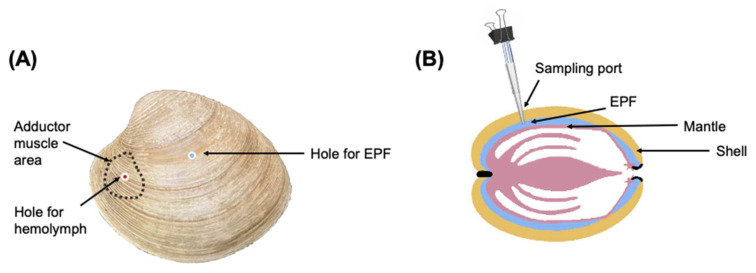
(**A**) Holes for accessing hemolymph and EPF. (**B**) Installation of sampling port.

A subsample of clams (3 clams from each bucket/12 clams per *p*CO_2_ treatment) was selected randomly to monitor the pHs of EPF and hemolymph over a period of 12 h. Holes were drilled in the shell of each clam using a round dental burr, avoiding damaging the mantle tissue. One hole was made to access the EPF directly in the center of the left valve (Figure 5) and a second hole was made over the anterior adductor muscle (Figure 5). To create an easily accessible “sampling port” [80], cut pipette tips (1000 μL) were inserted into the holes and then adhered in place with aquarium grade silicone (Figure 5). Vinyl airline tubing was attached to the top of the pipette tip. The tubing, which allowed for resampling of the fluid, was pinched using a binder clip when not in use to prevent exchange with external seawater. To account for potential stress, the clams were returned to their treatments for one week before sampling pH_e_. Prior to sampling, daily pH fluctuations were monitored to select the best time points to see diel changes. On the day of sampling, a pH microelectrode (Ohaus; Parsippany, NJ, USA) was inserted into the pipette tip until the sensing part of the electrode was covered with the fluid. For hemolymph (and in cases in which not enough EPF was drawn into the pipette tip), a syringe was used to create a vacuum and draw fluid into the tube. The micro pH probe was calibrated with pH 4.01, 7.0, and 10.01 NBS buffers (Thermo Scientific ™ Orion ™ Standard All-in-One pH Buffer Kit; Waltham, MA, USA) prior to each time point. When readings stabilized, pH was recorded. Sampling was conducted at three time points over a 12 h period (6:00 AM, 12:00 PM, and 6:00 PM) based on pH data for seawater measured in the previous days to reflect diel changes. Due to the invasive nature of this procedure, these clams were excluded from future sampling.

### 4.4. EPF and Hemolymph Collection 

EPF fluid and hemolymph samples were individually collected from 40 clams (five per tank, 4 tanks, equating to a total of 20 clams per *p*CO_2_ treatment), following the protocols outlined in Schwaner et al. (2022) [23]. Using a round dental burr, a hole was drilled in the center of the left valve for EPF extraction (Figure 5). If EPF volume was <1 mL, EPF was collected from both valves of each clam and pooled, so that the quantity of EPF was sufficient for downstream analyses. To access hemolymph, a second hole was drilled (Figure 5) over the anterior adductor muscle and hemolymph was extracted using a syringe. Approximately 1–1.5 mL of each fluid type was collected. EPF sample quality was assessed by examination of mantle integrity after opening the valves, and if the mantle was punctured, a new clam was sampled. After measuring pH, 100 µL was collected from each fluid type for hemocyte functional assays and diluted 1:4 with ice-cold filtered artificial seawater (FASW 30 PSU). The remaining fluid (1.4–0.9 mL) was centrifuged (800 g, 4 °C, 10 min). The supernatant was transferred to a new collection tube and protease inhibitor cocktail (SIGMA*FAST*^TM^ Protease Inhibitor Tablets; 50 µL of 1× solution prepared according to the manufacturer’s recommendations added to 1 mL of fluid; Sigma-Aldrich, Inc., St. Louis, MO, USA) was added. The pelleted cells and tubes containing supernatant were flash frozen and stored at −80 °C.

### 4.5. Histology and qPCR

After fluid samples were collected and the clams were shucked to check the integrity of the mantle tissue, the soft bodies were dissected for histopathology. A cross section of the body, along with diagonal pieces of the mantle from each valve, and a piece of siphon were collected and added to cassettes and fixed in formalin. Tissues were embedded in paraffin wax, sectioned (5 µM thick), mounted on slides, and stained with hematoxylin and eosin. Slides were read on a compound light microscope to look for *M. quahogii* (formerly QPX, a common pathogen of the hard clam) and any other pathological conditions. During dissection, if mantle tissue contained nodules (a usual sign of *M. quahogii* disease), qPCR was performed to check for *M. guahogii* infection. In these cases, DNA was extracted using a DNeasy Blood and Tissue Kit (Qiagen), following the manufacturer’s instructions, and qPCR was performed on an Applied Biosystems^TM^ QuantStudio^TM^ 6 Flex Real-Time PCR system, following an open access protocol outlined in Geraci Yee et al. (2022) “Supplement 3 methods protocol: Procedure for QPX qPCR Assay for Clam Tissue Samples” [81] (primers in Table S8).

### 4.6. Functional Hemocyte Assays

Flow cytometry (BD FACSCalibur) was used to assess hemocyte activities as described in Schwaner et al. (2022) [23]. Fluorescent signals emitted by hemocytes following the addition of dyes that target specific pathways or molecules were measured at a minimum of 1000 hemocytes. Cells were separated from debris by size and intracellular complexity, which is a standard procedure for bivalves that does not require the addition of specific dyes that could otherwise alter cellular activities [82,83]. Agranulocytes and granulocytes (sub-populations of hemocytes) were separated based on light forward (FSC) and side (SSC) scatter parameters and treated separately for downstream analyses [82].

Viability: Propidium iodide (PI; Thermo Fisher Scientific, Waltham, MA, USA) was added at a final concentration of 20 μg/mL and incubated for 10 min in the dark at room temperature (RT) before flow cytometry readings. PI only binds to DNA in dead cells, consequently making dead cells fluorescent in the orange (FL2) channel.

Intracellular pH: Fluid samples were transferred to sealed 0.5 mL microcentrifuge tubes to minimize gas exchange and then immediately centrifuged (800 g, 4 °C, 10 min). The supernatant was removed and the pellet was resuspended in FASW containing 2′,7′-bis-(2-carboxyethyl)-5-(and-6)-carboxyfluorescein, acetoxymethyl ester (BCECF-AM; Sigma, St. Louis, MO, USA) at a final concentration of 1 μM, and incubated at RT in the dark for 10 min. This dye shows an increase in green fluorescence (FL1 channel) intensity with increasing levels of pH. 

Calcium measurements: Relative Ca^2+^ contents in hemocytes were assessed using Fluo-3 (Thermo Fisher Scientific, Waltham, MA, USA), a dye that shows an increase in green fluorescence (FL1 channel) intensity with increasing levels of Ca^2+^. Fluo-3 was added at a final concentration of 5 μM and incubated at RT in the dark for 20 min before sample reading.

Phagocytosis: Yellow-green latex beads (2 μm; Sigma, St. Louis, MO, USA) were added to samples (1:10 hemocyte:bead ratio) and incubated at RT for 1 h before sample reading. Hemocytes associated with beads were considered phagocytic.

### 4.7. Statistical Analyses

All statistical analyses were performed in R (v. 4.2.0) using the graphical user interface Rstudio. The normal distribution and homoscedasticity of each data set were verified before applying parametric tests. Significant differences between the pHs of the seawater (control and OA) and clam fluids (EPF and hemolymph) were determined using Student’s *t*-tests at each time point investigated. EPF and hemolymph pHs from clams reared under OA conditions were compared with those of their corresponding controls maintained under normal conditions, also using Student’s *t*-tests. For functional assays, data were compared between EPF and hemolymph samples from different *p*CO_2_ conditions using nested ANOVAs. Data were generated from a total of 40 clams, with five individual clams sampled from four replicate tanks per *p*CO_2_ condition (n = 4). Differences were considered significant at *p* < 0.05.

### 4.8. RNA Extraction, Library Preparation, Sequencing, and Analysis 

RNA was extracted using TRIzol Reagent (Invitrogen, Thermo Fisher Scientific, Waltham, MA, USA; [84]). DNA was removed using a DNA-free^TM^ Kit (Ambion, Austin, TX, USA), following the manufacturer’s instructions. After checking the quality and quantity of RNA (Nanodrop, Thermo Fisher Scientific, Waltham, MA, USA), samples derived from EPF and hemolymph from the same eight clams were selected for sequencing. Extracted RNA was sent for sequencing to the Novogene Corporation (UC Davis, Sacramento, CA, USA). One microgram of RNA per sample was used as input material. Sequencing libraries were generated using a NEBNext^®^ Ultra^TM^ RNA Library Prep Kit for Illumina^®^ (NEB, Ipswich, MA, USA), following the manufacturer’s instructions and with indices added for the demultiplexing of samples. Libraries were sequenced on the Illumina platform (Novaseq 6000) and 150 paired-end (PE) reads were generated. Novogene performed quality control tests, and cleaned reads were used in downstream analyses. Cleaned sequence reads were trimmed based on quality scores (limit 0.05), ambiguous nucleotides (max 2 nucleotides per sequence), and adapters (CLC workbench (version 11.0.1)). Trimmed reads were mapped onto the *M. mercenaria* gene prediction [85] using the “map reads to reference” function with default parameters. The next in silico analyses were performed on the computing cluster SeaWulf at Stony Brook University. The reads were sorted, indexed, and the number of reads mapping to transcripts was quantified using Samtools version 1.9 [86]. Counts were normalized and compared between conditions (EPF OA vs. EPF control and hemolymph OA vs. hemolymph control) using the package *DESeq2* from Bioconductor [87] to perform differential gene expression analysis. Significant differences in gene expression between EPF and hemolymph in clams from different *p*CO_2_ treatments were identified with a cut-off threshold of adjusted *p*-values < 0.05 after Benjamini–Hochberg correction for multiple comparisons and log2fold changes > |2|. Hierarchical clustering heat maps were generated for each comparison for the top differentially expressed genes (DEGs). The functional annotation of *M. mercenaria* proteins generated from the gene annotation [85] was used. A hypergeometric test was performed to test for statistical enrichment (Benjamini–Hochberg correction to account for multiple comparisons to obtain an adjusted *p*-value < 0.05) of Gene Ontology (GO) terms using the R package *TopGo* [88]. Gene co-expression network analysis was performed using the R package *WGCNA* [89]. This was performed to identify and cluster genes that were co-expressed among the individual clams to determine whether OA causes changes in gene expression in response to OA that might be missed by gene-level differential expression analysis. A weighted gene correlation networks for analysis (WGCNA) pipeline was followed based on tutorials by Horvath and Langfelder (2011) [90] and Chang et al. (2021) [91] and supplementary information from Downey-Wall et al. (2020) [18]. Briefly, modules were created using the *adjacency*, *TOMsimilarity*, *hclust*, and *cutreeDynamic* functions in R with a minimum gene membership threshold of 30. Eigenvalues for module expression were calculated using *moduleEigengene*s. Linear regressions were performed to identify modules that were associated with EPF change in pH_i_ and hemolymph pH_i_ and intracellular calcium. Modules were investigated for the enrichment of GO categories as described previously. 

### 4.9. Proteomics on Cell-Free EPF and Plasma

Proteomics analysis was performed following the protocol in Schwaner et al. (2022) [23]. Cell-free EPF and hemolymph (i.e., plasma) were solubilized in 5% SDS, 100 mM TEAB, 10 mM DTT, at 55 °C for 30 min. Reduced cysteines were alkylated with 20 mM iodoacetamide for 30 min at RT in the dark, and proteins were acidified with phosphoric acid. Then, proteins were micro-precipitated with 90% methanol, 50mM TEAB, and bound to S-Trap solid-phase cartridges as described elsewhere [92]. Protein precipitates were washed with 90% methanol, 50 mM TEAB, and digested with trypsin at 47 °C for two hours before elution with sequential 50 mM TEAB, 0.2% formic acid and 50% acetonitrile (ACN), 0.2% formic acid elution steps by centrifugation (4000× *g* 1 min). Peptides were analyzed by C18 reverse phase LC-MS/MS. HPLC C18 columns were prepared using a P-2000 CO2 laser puller (Sutter Instruments) and silica tubing (100 µM ID × 20 cm) and were self-packed with 3u Reprosil resin. Peptides were separated using a flow rate of 300 nl/min, and a gradient elution step changing from 0.1% formic acid to 40% ACN over 90 min, followed 90% ACN wash and re-equilibration steps. Parent peptide mass and collision-induced fragment mass information were collected using an orbital trap (Q-Exactive HF; Thermo Fisher Scientific, Waltham, MA, USA) instrument, followed by protein database searching using Proteome Discoverer 2.4 (Thermo Fisher Scientific, Waltham, MA, USA). Electrospray ionization was achieved using a spray voltage of ~2.2 kV. Information-dependent MS and MS-MS acquisitions were made using a 100 ms survey scan (*m*/*z* 375–1400) at 60,000 resolution, followed typically by “top 20” consecutive second product ion scans at 15,000 resolution. For the database search, Proteome Discoverer 2.4 (Thermo Fisher Scientific, Waltham, MA, USA) was used. Peptide and spectra false discovery rates were set to 0.05. Sample normalization was based on total peptide amount. Label-free quantitation (LFQ) between samples was performed using intensity-based pairwise like-peptide comparisons to generate fold-change ratios. Protein abundance was based on summed pairwise peptide abundances and Student’s *t*-tests. Shared and modified peptides (oxidated-M, deamidated-NQ, dehydrated-ST) were excluded from quantitation. The resultant proteins were mapped to the predicted proteins annotated from the whole genome [85]. Protein expression was compared between EPF OA vs. control and hemolymph OA vs. control and filtered for log2foldchanges >|0.8| and adjusted *p*-values < 0.05. GO analysis was performed as previously described.

## 5. Conclusions

This integrated analysis of the physiological and molecular changes in calcifying fluids in response to OA demonstrated that *M. mercenaria* have the capacity to control pH and maintain biomineralization via the regulation of biomineralization-, acid–base-balance-, and calcium-related genes in their EPF and hemolymph. However, there could be trade-offs between immune and biomineralization functions of hemocytes. Several genes that appear to play key roles in the maintenance of biomineralization have been identified at the mRNA and/or protein level, although further research using functional genomic approaches needs to be conducted to confirm the precise roles of target genes (e.g., using gene knockdown). Overall, we conclude that *M. mercenaria* can promote biomineralization and maintain acid–base balance under OA; however, this might come with physiological trade-offs that could make them more vulnerable to disease or other environmental stressors.

## Figures and Tables

**Table 1:** Differentially expressed genes in EPF and hemolymph from clams in OA vs. clams in control conditions. Expression level corresponds to the OA treatment. Log2foldchanges (LFC) and adjusted *p*-values (padj) for each DEG are given.

ID	Description	Extrapallial Fluid		Hemolymph	
		LFC	Padj	LFC	Padj
mRNA.chromosome_2.1379.1	Protocadherin Fat 1/2/3	−8.43	<0.001	−8.2	<0.001
mRNA.chromosome_17.1242.1	Integrase catalytic domain profile	−6.22	<0.001	−5	<0.001
mRNA.chromosome_12.417.1	Cathepsin L	−5.93	<0.001		
mRNA.chromosome_3.570.1	Glutamate receptor ionotropic			−5.55	0.04
mRNA.chromosome_19.1763.2	Membrane alanyl dipeptidase (M1) family signature			−5.49	0.05
mRNA.chromosome_11.788.1	Glutamate receptor ionotropic			−5.23	0.04
mRNA.chromosome_9.119.1	Protocadherin Fat 1/2/3	−5.16	0.001	−5.92	<0.001
mRNA.chromosome_8.169.3	Golgin subfamily B member 1	−4.97	0.001		
mRNA.contig_845.1.1	Pol-like protein	−4.25	0.03		
mRNA.contig_1983.1.1	Tumor necrosis factor family	−4.08	0.05		
mRNA.chromosome_16.1283.1	Integrase catalytic domain profile	−3.94	0.04	−4.54	<0.001
mRNA.chromosome_8.1919.2	Scavenger receptor cysteine-rich type 1 protein	−3.46	<0.01		
mRNA.chromosome_2.418.1	Thrombospondin type-1 (TSP1) repeat profile	−3.29	<0.01		
mRNA.chromosome_11.1914.1	Tubulin alpha	−3.15	0.04		
mRNA.chromosome_10.859.1	Zinc finger protein 862-like isoform	−3.08	<0.01		
mRNA.chromosome_4.2461.1	Uncharacterized	−2.24	<0.01		
mRNA.chromosome_17.1557.1	Sulfotransferase domain			2.09	0.03
mRNA.chromosome_7.1244.1	Cytochrome P450			2.12	<0.01
mRNA.chromosome_17.196.2	Carboxylesterases type-B serine active site	2.16	<0.01		
mRNA.chromosome_2.915.1	Carboxylesterases type-B signature 2	2.19	<0.001	2.19	<0.001
mRNA.chromosome_17.1090.1	Cytochrome P450	2.19	0.03		
mRNA.chromosome_11.1022.3	ATP-binding cassette, subfamily B	2.24	0.04		
mRNA.chromosome_7.1826.1	Phospholipid-translocating ATPase	2.82	0.01		
mRNA.contig_573.4.1	Uncharacterized	2.87	0.04		
mRNA.chromosome_13.1522.1	Fatty acid synthase, animal type	2.9	0.02		
mRNA.chromosome_18.134.1	Integrase catalytic domain profile			2.97	0.05
mRNA.chromosome_16.500.1	Acyl-CoA dehydrogenase, middle domain	3.08	0.05		
mRNA.chromosome_4.2319.1	Cytochrome P450			3.21	0.03
mRNA.chromosome_7.2111.1	Solute carrier family 25	3.65	<0.001		
mRNA.chromosome_5.470.1	G protein beta WD-40 repeat signature	3.82	0.04		
mRNA.chromosome_12.413.4	Solute carrier family 6	4.37	0.04		
mRNA.chromosome_13.2224.1	Soluble glutathione S-transferase C-terminal domain profile			4.54	0.05
mRNA.chromosome_18.1048.1	Myosin-3-like	4.82	0.02	6.11	0.02
mRNA.chromosome_4.1870.1	Brinker DNA-binding domain	4.97	0.04		
mRNA.chromosome_15.405.1	Folylpolyglutamate synthase signature 1	5.15	<0.01		
mRNA.contig_3725.2.1	RNA-directed DNA polymerase from transposon BS	5.85	0.02		
mRNA.chromosome_18.1047.1	Myosin-3-like	5.96	0.001	6.57	<0.001
mRNA.chromosome_3.951.1	Interferon-induced helicase C domain-containing protein 1	5.98	0.02		
mRNA.chromosome_16.1831.1	Uncharacterized	6.3	0.03		
mRNA.chromosome_17.2204.1	Theromacin	9.18	0.01	12.98	<0.001
mRNA.chromosome_17.2202.1	Theromacin	9.45	0.03	14.17	<0.001

**Table 2:** Genes showing differential expression at the protein level in both EPF and hemolymph from clams in OA vs. clams in control conditions. Expression level corresponds to the OA treatment. Log2foldchange (LFC) is given. The table is divided into subsections by proteins, with similar trends in both fluids and then opposing trends.

		Extrapallial Fluid	Hemolymph
ID	Description	LFC	LFC
mRNA.chromosome_9.771.1	Ficolin	3.32	3.32
mRNA.chromosome_1.367.2	20S proteasome subunit alpha 1	3.32	3.32
mRNA.chromosome_15.2247.1	Uncharacterized	3.32	3.32
mRNA.chromosome_11.2118.1	Sarcoplasmic calcium binding protein	3.32	3.32
mRNA.chromosome_13.2773.1	Chitin-binding type-2 domain profile	3.32	3.32
mRNA.chromosome_13.2650.1	Acetyltransferase (GNAT) family	3.32	3.32
mRNA.chromosome_10.1356.3	Peroxin-19	3.32	3.32
mRNA.chromosome_18.297.1	Titin-like	3.32	3.32
mRNA.chromosome_9.1438.1	Uncharacterized	3.32	3.32
mRNA.chromosome_13.636.1	Guanine nucleotide-binding protein G(i) subunit alpha	3.32	3.32
mRNA.chromosome_3.1719.1	Octopine dehydrogenase, opine dehydrogenase, tauropine dehydrogenase	3.32	3.32
mRNA.chromosome_13.1579.2	Transforming growth factor-beta-induced protein	3.02	3.32
mRNA.chromosome_5.1812.1	Transcription factor AP-4	2.84	2.96
mRNA.chromosome_19.1568.1	Alpha-2-macroglobulin 1	−1.94	−3.32
mRNA.chromosome_6.1724.1	20S proteasome subunit beta 5	−2.11	−3.02
mRNA.chromosome_3.2176.1	Nucleoprotein TPR	−2.11	−3.32
mRNA.chromosome_3.702.1	Mannose receptor, C type; C-type lectin domain profile	−2.38	−1.89
mRNA.chromosome_15.1157.1	N-acetylglucosamine-1-phosphotransferase subunits alpha/beta-like	−2.51	−2.61
mRNA.chromosome_8.1594.1	Universal stress protein signature	−3.12	−3.32
mRNA.chromosome_16.1148.1	Elongation factor 1-alpha-like	−3.32	−2.63
mRNA.chromosome_15.1817.1	Calcium-binding EGF domain	−3.32	−3.32
mRNA.chromosome_9.1938.1	Cell migration-inducing and hyaluronan binding protein	−3.32	−3.32
mRNA.chromosome_4.1618.1	Modulator of levamisole receptor-1	−3.32	−2.40
mRNA.chromosome_16.407.1	Complement C1q-like protein 2	−3.32	−3.32
mRNA.chromosome_10.109.1	Complement C1q tumor necrosis factor-related protein 3-like	−3.32	−2.41
mRNA.chromosome_13.2638.1	Dystroglycan 1	−3.32	−3.32
mRNA.chromosome_19.2475.1	Complement C1q tumor necrosis factor-related protein 2-like	−3.32	−3.32
mRNA.chromosome_10.1347.1	Fatty acid-binding protein	−3.32	−3.32
mRNA.chromosome_7.148.3	UDP-glucose:glycoprotein glucosyltransferase	−3.32	−3.32
mRNA.chromosome_4.2588.1	EF-hand calcium-binding domain profile	−3.32	−3.32
mRNA.chromosome_10.1861.2	3-hydroxyisobutyryl-CoA hydrolase	−3.32	−3.32
mRNA.chromosome_3.1856.1	Pancreatic triacylglycerol lipase	−3.32	−2.86
mRNA.chromosome_12.1120.1	Endoplasmic reticulum protein 29	3.32	−3.32
mRNA.chromosome_4.2111.1	Peroxiredoxin (alkyl hydroperoxide reductase subunit C)	3.32	−3.32
mRNA.chromosome_13.726.1	Sodium/calcium exchanger regulatory protein 1-like	3.27	−3.32
mRNA.chromosome_7.1691.1	Laminin, alpha 3/5	2.82	−3.32
mRNA.chromosome_5.3377.1	Mannose receptor; Lectin C-type domain	1.83	−3.32
mRNA.chromosome_18.1635.1	Fibrinogen C-terminal domain profile	−3.32	3.32
mRNA.chromosome_16.1096.1	Ficolin-1-like	−3.32	3.32
mRNA.chromosome_10.78.1	Trichohyalin	−3.32	3.32
mRNA.chromosome_2.1431.4	Calcium-binding EGF domain	−3.32	2.52

## Data Availability

The original contributions presented in the study are publicly available. Transcriptomics data can be found at https://www.ncbi.nlm.nih.gov/sra under the accession numbers SAMN31684258-SAMN31684289. Proteomics data can be found at https://massive.ucsd.edu/ProteoSAFe/static/massive.jps.

## Supplementary Materials

The following supporting information can be downloaded at: https://www.mdpi.com/article/10.3390/ijms232416066/s1, Figure S1: Suspicious nodule detected in the mantle of *M. mercenaria* maintained in high-*p*CO_2_ conditions. Nodules are often associated with infection by *Mucochytrium quahogii*, although this clam was negative for the parasite; Table S1: DEGs in hemocytes from EPF collected from clams in OA vs. clams in control conditions. Expression level corresponds to the OA treatment; Table S2: DEGs in hemocytes from hemolymph collected from clams in OA vs. clams in control conditions. Expression level corresponds to the OA treatment; Table S3: Excel file. Gene co-expression network analysis of hemocytes from EPF identified six modules of co-expressed genes; Table S4: Excel file. Gene co-expression network analysis of hemocytes from hemolymph identified six modules of co-expressed genes; Table S5: Excel file. Differentially expressed proteins of cell-free EPF from clams in OA vs. clams in control conditions. Expression level corresponds to the OA treatment; Table S6: Excel file. Differentially expressed proteins of plasma (cell-free hemolymph) from clams in OA vs. clams in control conditions. Expression level corresponds to the OA treatment; Table S7: Seawater chemistry; Table S8: Primers used for the QPX qPCR assay (produced by Integrated DNA Technologies, Coralville, Iowa).

## References

[1] Doney S.C., Fabry V.J., Feely R.A., Kleypas J.A. (2009). Ocean acidification: The other CO_2_ problem. Annu. Rev. Mar. Sci..

[2] Feely R.A., Doney S.C., Cooley S.R. (2009). Ocean acidification: Present conditions and future changes in a high-CO_2_ world. Oceanography.

[3] Wallace R.B., Baumann H., Grear J.S., Aller R.C., Gobler C.J. (2014). Coastal ocean acidification: The other eutrophication problem. Estuar. Coast. Shelf Sci..

[4] Guinotte J.M., Fabry V.J. (2008). Ocean acidification and its potential effects on marine ecosystems. Ann. N. Y. Acad. Sci..

[5] Welladsen H.M., Southgate P.C., Heimann K. (2010). The effects of exposure to near-future levels of ocean acidification on shell characteristics of *Pinctada fucata* (Bivalvia: Pteriidae). Molluscan Res..

[6] Feely R.A., Alin S.R., Carter B., Bednaršek N., Hales B., Chan F., Hill T.M., Gaylord B., Sanford E., Byrne R.H. (2016). Chemical and biological impacts of ocean acidification along the west coast of North America. Estuar. Coast. Shelf Sci..

[7] National Marine Fisheries Service Office of Science and Technology (2018). Fisheries of the United States.

[8] Cerrato R., Caron D., Lonsdale D., Rose J., Schaffner R. (2004). Effect of the northern quahog *Mercenaria mercenaria* on the development of blooms of the brown tide alga *Aureococcus anophagefferens*. Mar. Ecol. Prog..

[9] Kroeker K.J., Kordas R.L., Crim R.N., Singh G.G. (2010). Meta-analysis reveals negative yet variable effects of ocean acidification on marine organisms. Ecol. Lett..

[10] Hall-Spencer J.M., Thorndyke M., Dupont S. (2015). Impact of ocean acidification on marine organisms—Unifying principles and new paradigms. Water.

[11] Baumann H., Wallace R.B., Tagliaferri T., Gobler C.J. (2015). Large natural pH, CO_2_ and O_2_ fluctuations in a temperate tidal salt marsh on diel, seasonal, and interannual time scales. Estuaries Coasts.

[12] Crenshaw M.A. (1972). The inorganic composition of molluscan extrapallial fluid. Biol. Bull..

[13] Ramesh K., Hu M.Y., Thomsen J., Bleich M., Melzner F. (2017). Mussel larvae modify calcifying fluid carbonate chemistry to promote calcification. Nat. Comm..

[14] Wang X., Wang M., Jia Z., Wang H., Jiang S., Chen H., Wang L., Song L. (2016). Ocean acidification stimulates alkali signal pathway: A bicarbonate sensing soluble adenylyl cyclase from oyster *Crassostrea gigas* mediates physiological changes induced by CO_2_ exposure. Aquat. Toxicol..

[15] Wang X., Wang M., Jia Z., Qiu L., Wang L., Zhang A., Song L. (2017). A carbonic anhydrase serves as an important acid-base regulator in pacific oyster *Crassostrea gigas* exposed to elevated CO_2_: Implication for physiological responses of mollusk to ocean acidification. Mar. Biotechnol..

[16] Michaelidis B., Ouzounis C., Paleras A., Pörtner H.O. (2005). Effects of long-term moderate hypercapnia on acid-base balance and growth rate in marine mussels *Mytilus galloprovincialis*. Mar. Ecol. Prog. Ser..

[17] Auzoux-Bordenave S., Chevret S., Badou A., Martin S., Di Giglio S., Dubois P. (2021). Acid–base balance in the hæmolymph of European abalone (*Haliotis tuberculata*) exposed to CO_2_-induced ocean acidification. Comp. Biochem. Physiol..

[18] Downey-Wall A.M., Cameron L.P., Ford B.M., McNally E.M., Venkataraman Y.R., Roberts S.B., Ries J.B., Lotterhos K.E. (2020). Ocean acidification induces subtle shifts in gene expression and DNA methylation in mantle tissue of the Eastern oyster (*Crassostrea virginica*). Front. Mar. Sci..

[19] Zhao X., Shi W., Han Y., Liu S., Guo C., Fu W., Chai X., Liu G. (2017). Ocean acidification adversely influences metabolism, extracellular pH and calcification of an economically important marine bivalve, *Tegillarca granosa*. Mar. Environ. Res..

[20] Zittier Z.M., Bock C., Lannig G., Pörtner H.O. (2015). Impact of ocean acidification on thermal tolerance and acid–base regulation of *Mytilus edulis* (L.) from the North Sea. J. Exp. Mar. Biol. Ecol..

[21] Gazeau F., Alliouane S., Bock C., Bramanti L., López Correa M., Gentile M., Hirse T., Pörtner H.O., Ziveri P. (2014). Impact of ocean acidification and warming on the Mediterranean mussel (*Mytilus galloprovincialis*). Front. Mar. Sci..

[22] Mount A.S., Wheeler A.P., Paradkar R.P., Snider D. (2004). Hemocyte-Mediate Shell Mineralization in the Eastern Oyster. Science.

[23] Schwaner C., Farhat S., Haley J., Espinosa E.P., Allam B. (2022). Transcriptomic, Proteomic, and Functional Assays Underline the Dual Role of Extrapallial Hemocytes in Immunity and Biomineralization in the Hard Clam *Mercenaria mercenaria*. Front. Immunol..

[24] Wood H.L., Spicer J.I., Widdicombe S. (2008). Ocean acidification may increase calcification rates, but at a cost. Proc. R. Soc. B.

[25] Huang J., Li S., Liu Y., Liu C., Xie L., Zhang R. (2018). Hemocytes in the extrapallial space of *Pinctada fucata* are involved in immunity and biomineralization. Sci. Rep..

[26] Bibby R., Widdicombe S., Parry H., Spicer J., Pipe R. (2008). Effects of ocean acidification on the immune response of the blue mussel *Mytilus edulis*. Aquat. Biol..

[27] Sun T., Tang X., Jiang Y., Wang Y. (2017). Seawater acidification induced immune function changes of haemocytes in *Mytilus edulis*: A comparative study of CO_2_ and HCl enrichment. Sci. Rep..

[28] Liu S., Shi W., Guo C., Zhao X., Han Y., Peng C., Chai X., Liu G. (2016). Ocean acidification weakens the immune response of blood clam through hampering the NF-kappa β and toll-like receptor pathways. Fish Shellfish Immunol..

[29] Cao R., Wang Q., Yang D., Liu Y., Ran W., Qu Y., Wu H., Cong M., Li F., Ji C. (2018). CO_2_-induced ocean acidification impairs the immune function of the Pacific oyster against *Vibrio splendidus* challenge: An integrated study from a cellular and proteomic perspective. Sci. Total Environ..

[30] Schwaner C., Barbosa M., Connors P., Park T., Silva D., Griffith A., Gobler C.J., Pales E., Allam B. (2020). Experimental acidification increases susceptibility of *Mercenaria mercenaria* to infection by *Vibrio* species. Mar. Environ. Res..

[31] Todgham A.E., Hofmann G.E. (2009). Transcriptomic response of sea urchin larvae *Strongylocentrotus purpuratus* to CO_2_-driven seawater acidification. J. Exp. Biol..

[32] Di G., Li Y., Zhu G., Guo X., Li H., Huang M., Shen M., Ke C. (2019). Effects of acidification on the proteome during early development of *Babylonia areolata*. FEBS Open Bio.

[33] Dineshram R., Sharma R., Chandramouli K., Yalamanchili H.K., Chu I., Thiyagarajan V. (2015). Comparative and quantitative proteomics reveal the adaptive strategies of oyster larvae to ocean acidification. Proteomics.

[34] Johnson K.M., Hofmann G.E. (2017). Transcriptomic response of the Antarctic pteropod *Limacina helicina* antarctica to ocean acidification. BMC Genom..

[35] Chandra Rajan K., Meng Y., Yu Z., Roberts S.B., Vengatesen T. (2021). Oyster biomineralization under ocean acidification: From genes to shell. Glob. Chang. Biol..

[36] Thomsen J., Melzner F. (2010). Moderate seawater acidification does not elicit long-term metabolic depression in the blue mussel *Mytilus edulis*. Mar. Biol..

[37] Lannig G., Eilers S., Pörtner H.O., Sokolova I.M., Bock C. (2010). Impact of ocean acidification on energy metabolism of oyster, *Crassostrea gigas*—Changes in metabolic pathways and thermal response. Mar. Drugs.

[38] Heisler N. (1989). Interactions between gas exchange, metabolism, and ion transport in animals: An overview. Can. J. Zool..

[39] Abe H. (2000). Role of histidine-related compounds as intracellular proton buffering constituents in vertebrate muscle. Biochem. C/C Biokhimiia.

[40] Melzner F., Mark F.C., Seibel B.A., Tomanek L. (2020). Ocean Acidification and Coastal Marine Invertebrates: Tracking CO_2_ Effects from Seawater to the Cell. Ann. Rev. Mar. Sci..

[41] Zhao L., Milano S., Walliser E.O., Schöne B.R. (2018). Bivalve shell formation in a naturally CO_2_-enriched habitat: Unraveling the resilience mechanisms from elemental signatures. Chemosphere.

[42] Widdicombe S., Spicer J.I. (2008). Predicting the impact of ocean acidification on benthic biodiversity: What can animal physiology tell us?. J. Exp. Mar. Biol..

[43] Ivanina A.V., Falfushynska H.I., Beniash E., Piontkivska H., Sokolova I.M. (2017). Biomineralization-related specialization of hemocytes and mantle tissues of the Pacific oyster *Crassostrea gigas*. J. Exp. Biol..

[44] Wang X., Wang M., Wang W., Liu Z., Xu J., Jia Z., Chen H., Qiu L., Lv Z., Wang L. (2020). Transcriptional changes of Pacific oyster *Crassostrea gigas* reveal essential role of calcium signal pathway in response to CO_2_-driven acidification. Sci. Total Environ..

[45] Ivanina A.V., Borah B.M., Vogts A., Malik I., Wu J., Chin A.R., Almarza A.J., Kumta P., Piontkivska H., Beniash E. (2018). Potential trade-offs between biomineralization and immunity revealed by shell properties and gene expression profiles of two closely related *Crassostrea species*. J. Exp. Biol..

[46] Su W., Rong J., Zha S., Yan M., Fang J., Liu G. (2018). Ocean acidification affects the cytoskeleton, lysozymes, and nitric oxide of hemocytes: A possible explanation for the hampered phagocytosis in blood clams, *Tegillarca granosa*. Front. Physiol..

[47] Thomas C. (1996). Hemocytes: Forms and functions. East. Oyster Crassostrea Virginica.

[48] Wu F., Lu W., Shang Y., Kong H., Li L., Sui Y., Hu M., Wang Y. (2016). Combined effects of seawater acidification and high temperature on hemocyte parameters in the thick shell mussel *Mytilus coruscus*. Fish Shellfish Immunol..

[49] Allam B., Paillard C., Auffret M. (2000). Alterations in hemolymph and extrapallial fluid parameters in the Manila clam, *Ruditapes philippinarum* challenged with the pathogen, *Vibrio tapetis*. J. Invertebr. Pathol..

[50] Allam B., Ashton-Alcox K.A., Ford S.E. (2001). Haemocyte parameters associated with resistance against brown ring disease in clams. Dev. Comp. Immunol..

[51] Allam B., Raftos D. (2015). Immune responses to infectious diseases in bivalves. J. Invertebr. Pathol..

[52] Shen W., Hu Y., He Z., Xu S., Xu X., Zhang G., Ren G. (2020). Histological and Comparative Transcriptome Analyses Provide Insights Into the Immune Response in Pearl Sac Formation of *Hyriopsis cumingii*. Front. Mar. Sci..

[53] Gardner L.D., Mills D., Wiegand A., Leavesley D., Elizur A. (2011). Spatial analysis of biomineralization associated gene expression from the mantle organ of the pearl oyster *Pinctada maxima*. BMC Genom..

[54] Pereira-Mouriès L., Almeida M.J., Ribeiro C., Peduzzi J., Barthélemy M., Milet C. (2002). Soluble silk-like organic matrix in the nacreous layer of the bivalve *Pinctada maxima*. Eur. J. Biochem..

[55] Tasiemski A., Vandenbulcke F., Mitta G., Lemoine J., Lefebvre C., Sautiere P.E., Salzet M. (2004). Molecular characterization of two novel antibacterial peptides inducible upon bacterial challenge in an annelid, the leech *Theromyzon tessulatum*. J. Biol. Chem..

[56] Kim I.W., Morse D.E., Evans J.S. (2004). Molecular characterization of the 30-AA N-terminal mineral interaction domain of the biomineralization protein AP7. Langmuir.

[57] Timmins-Schiffman E., Coffey W.D., Hua W., Nunn B.L., Dickinson G.H., Roberts S.B. (2014). Shotgun proteomics reveals physiological response to ocean acidification in *Crassostrea gigas*. BMC Genom..

[58] Zhao X., Han Y., Chen B., Xia B., Qu K., Liu G. (2014). CO_2_-driven ocean acidification weakens mussel shell defense capacity and induces global molecular compensatory responses. Chemosphere.

[59] Carroll S.L., Coyne V.E. (2021). A proteomic analysis of the effect of ocean acidification on the haemocyte proteome of the South African abalone *Haliotis midae*. Fish Shellfish Immunol..

[60] Cordat E., Casey J.R. (2009). Bicarbonate transport in cell physiology and disease. Biochem. J..

[61] Goncalves P., Thompson E.L., Raftos D.A. (2017). Contrasting impacts of ocean acidification and warming on the molecular responses of CO_2_-resilient oysters. BMC Genom..

[62] Arivalagan J., Yarra T., Marie B., Sleight V.A., Duvernois-Berthet E., Clark M.S., Marie A., Berland S. (2017). Insights from the shell proteome: Biomineralization to adaptation. Mol. Biol. Evol..

[63] Henry R.P., Cameron J.N. (1983). The role of carbonic anhydrase in respiration, ion regulation and acid-base balance in the aquatic crab *Calunectes sapidus* and the terrestrial crab *Gecarcinus lateraus*. J. Exp. Biol..

[64] Ramesh K., Hu M.Y., Melzner F., Bleich M., Himmerkus N. (2020). Intracellular pH regulation in mantle epithelial cells of the Pacific oyster, *Crassostrea gigas*. J. Comp. Physiol..

[65] Hüning A.K., Melzner F., Thomsen J., Gutowska M.A., Krämer L., Frickenhaus S., Rosenstiel P., Pörtner H.-O., Philipp E.E.R., Lucassen M. (2013). Impacts of seawater acidification on mantle gene expression patterns of the Baltic Sea blue mussel: Implications for shell formation and energy metabolism. Mar. Biol..

[66] Liu Z., Zhang Y., Zhou Z., Zong Y., Zheng Y., Liu C., Kong N., Gao Q., Wang L., Song L. (2020). Metabolomic and transcriptomic profiling reveals the alteration of energy metabolism in oyster larvae during initial shell formation and under experimental ocean acidification. Sci. Rep..

[67] De Wit P., Durland E., Ventura A., Langdon C.J. (2018). Gene expression correlated with delay in shell formation in larval Pacific oysters (*Crassostrea gigas*) exposed to experimental ocean acidification provides insights into shell formation mechanisms. BMC Genom..

[68] Suzuki M., Kogure T., Nagasawa H. (2017). Studies on the chemical structures of organic matrices and their functions in the biomineralization processes of molluscan shells. AGri-Biosci. Monogr..

[69] Carreiro-Silva M., Cerqueira T., Godinho A., Caetano M., Santos R.S., Bettencourt R. (2014). Molecular mechanisms underlying the physiological responses of the cold-water coral *Desmophyllum dianthus* to ocean acidification. Coral Reefs.

[70] Servetto N., de Aranzamendi M.C., Bettencourt R., Held C., Abele D., Movilla J., González G., Sahade R. (2021). Molecular mechanisms underlying responses of the Antarctic coral *Malacobelemnon daytoni* to ocean acidification. Mar. Environ. Res..

[71] Pales Espinosa E., Koller A., Allam B. (2016). Proteomic characterization of mucosal secretions in the eastern oyster, *Crassostrea virginica*. J. Proteom..

[72] Allam B., Paillard C. (1998). Defense factors in clam extrapallial fluids. Dis. Aquat. Organ..

[73] Melzner F., Podbilski I., Mark F.C., Tresguerres M. (2022). The silent loss of cell physiology hampers marine biosciences. PLoS Biol..

[74] Pachauri R.K., Meyer L.A., IPCC, Core Writing Team (2014). Climate Change 2014: Synthesis Report. Contribution of Working Groups I, II and III to the Fifth Assessment Report of the Intergovernmental Panel on Climate Change.

[75] Caldeira K., Wickett M.E. (2003). Anthropogenic carbon and ocean pH. Nature.

[76] Cornwall C.E., Hurd C.L. (2016). Experimental design in ocean acidification research: Problems and solutions. ICES Mar. Sci..

[77] Gattuso J.P., Epitalon J.M., Lavigne H., Orr J. (2018). Seacarb: Seawater Carbonate Chemistry.

[78] Dickson A.G., Sabine C.L., Christian J.R. (2007). Guide to Best Practices for Ocean CO_2_ Measurements.

[79] Millero F.J. (2010). Carbonate constants for estuarine waters. Mar. Freshw. Res..

[80] Stemmer K., Brey T., Gutbrod M.S., Beutler M., Schalkhausser B., De Beer D. (2019). In situ Measurements of pH, CA^2+^, and Dic Dynamics within the Extrapallial Fluid of the Ocean Quahog *Arctica islandica*. J. Shellfish Res..

[81] Geraci-Yee S., Allam B., Collier J. (2022). Keeping up with advances in qPCR pathogen detection: An example for QPX disease in hard clams. Dis. Aquat. Org..

[82] Allam B., Ashton-Alcox K.A., Ford S.E. (2002). Flow Cytometric Characterization and Separation of Hemocytes from Three Bivalve Species. Fish Shellfish Immunol..

[83] Nguyen T.V., Alfaro C.A. (2019). Applications of Flow Cytometry in Molluscan Immunology: Current Status and Trends. Fish Shellfish Immunol..

[84] Rio D.C., Ares M., Hannon G.J., Nilsen T.W. (2010). Purification of RNA using TRIzol (TRI reagent). Cold Spring Harbor Protoc..

[85] Farhat S., Bonnivard E., Pales Espinosa E., Tanguy A., Boutet I., Guiglielmoni N., Flot J.F., Allam B. (2022). Comparative analysis of the *Mercenaria mercenaria* genome provides insights into the diversity of transposable elements and immune molecules in bivalve mollusks. BMC Genom..

[86] Li H., Handsaker B., Wysoker A., Fennell T., Ruan J., Homer N., Marth G., Abecasis G., Durbin R. (2009). The Sequence Alignment/Map Format and SAMtools. Bioinformatics.

[87] Love M.I., Huber W., Anders S. (2014). Moderated Estimation of Fold Change and Dispersion for RNA-Seq Data With Deseq2. Genome Biol..

[88] Alexa A., Rahnenfuhrer J. (2020). topGO: Enrichment Analysis for Gene Ontology.

[89] Langfelder P., Horvath S. (2008). WGCNA: An R package for weighted correlation network analysis. BMC Bioinform..

[90] Horvath S., Langfelder P. Tutorial for the WGCNA Package for R: III. Using Simulated Data to Evaluate the Different Module Detection Methods and Gene Screening Approaches. 6: Relating Modules and Module Eigengenes to External Data. https://horvath.genetics.ucla.edu/html/CoexpressionNetwork/Rpackages/WGCNA/Tutorials/Simulated-06-RelatingToExt.pdf.

[91] Chang J. Bioinformatics Workbook: WGCNA Gene Correlation Network Analysis: Network Analysis with WGCNA. https://bioinformaticsworkbook.org/tutorials/wgcna.html#gsc.tab=0.

[92] Zougman A., Wilson J.P., Banks E.R. (2020). A Simple Serum Depletion Method for Proteomics Analysis. Biotechniques.

