# Supplementary material for: Proteomic and Transcriptomic Responses Enable Clams to Correct the pH of Calcifying Fluids and Sustain Biomineralization in Acidified Environments
Source: Int J Mol Sci. 2022 Dec 16;23(24):16066. doi: 10.3390/ijms232416066 (PMC9781830; doi:10.3390/ijms232416066)
Supplement: Supplementary file 1 [file ijms-23-16066-s001.zip › ijms-2083283-supplementary/IJMS Paper 1 Supp Files_edited/IJMS supplmental Paper 1_edited_BA.pdf]

International Journal of
Molecular Sciences

MDPI

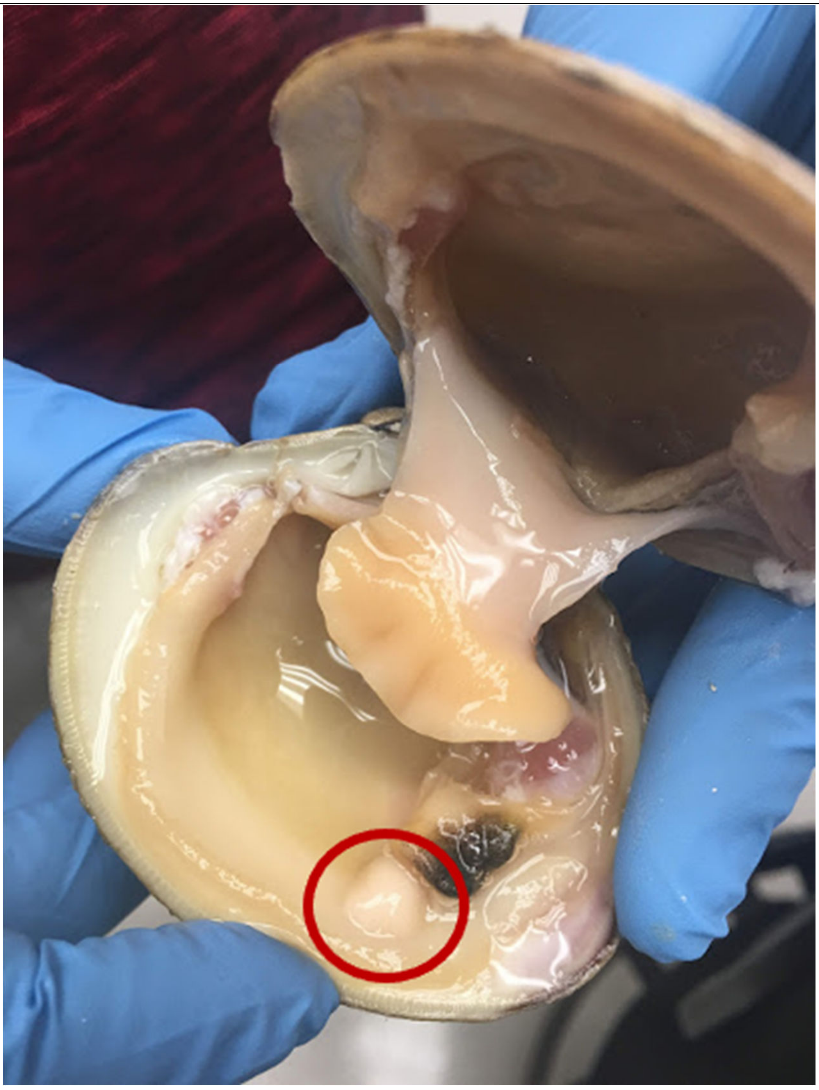

Figure S1. Suspicious nodule detected in the mantle of *M. mercenaria* maintained in high pCO_2 conditions. Nodules are often associated with infection by *Mucochytrium quahogii* although this clam was negative for the parasite.

Table S1. DEGs in hemocytes from EPF collected from clams in OA vs clams in control conditions. Expression level corresponds to the OA treatment.

Gene ID	Predicted Protein	L2FC	Adj p-value
mRNA.chromosome_2.1379.1	protocadherin Fat 1/2/3	-8.43	4.0E-06
mRNA.chromosome_17.1242.1	Integrase catalytic domain profile	-6.22	4.7E-07
mRNA.chromosome_12.417.1	cathepsin L	-5.93	1.0E-04
mRNA.chromosome_9.119.1	protocadherin Fat 1/2/3	-5.16	1.0E-03
mRNA.chromosome_8.169.3	golgin subfamily B member 1	-4.97	1.0E-03

mRNA.contig_845.1.1	pol-like protein	-4.25	2.7E-02
mRNA.contig_1983.1.1	TNF family profile	-4.08	5.1E-02
mRNA.chromosome_16.1283.1	Integrase catalytic domain profile.	-3.94	3.8E-02
mRNA.chromosome_8.1919.2	scavenger receptor cysteine-rich type 1 protein M160-like isoform X2	-3.46	7.4E-03
mRNA.chromosome_2.418.1	Thrombospondin type-1 (TSP1) repeat profile	-3.29	7.4E-03
mRNA.chromosome_11.1914.1	tubulin alpha	-3.15	3.9E-02
mRNA.chromosome_10.859.1	zinc finger protein 862-like isoform X1	-3.08	7.4E-03
mRNA.chromosome_4.2461.1	unnamed protein product	-2.24	7.4E-03
mRNA.chromosome_17.196.2	bile salt-activated lipase-like	2.16	2.8E-03
mRNA.chromosome_2.915.1	neuroligin	2.19	3.4E-06
mRNA.chromosome_17.1090.1	cytochrome P450 2B4-like	2.19	2.7E-02
mRNA.chromosome_11.1022.3	ATP-binding cassette, subfamily B	2.24	3.8E-02
mRNA.chromosome_7.1826.1	phospholipid-translocating ATPase	2.82	1.3E-02
mRNA.contig_573.4.1	--	2.87	3.7E-02
mRNA.chromosome_13.1522.1	fatty acid synthase, animal type	2.90	2.2E-02
mRNA.chromosome_16.500.1	Acyl-CoA dehydrogenase, middle domain	3.08	5.4E-02
mRNA.chromosome_7.2111.1	solute carrier family 25	3.65	4.0E-04
mRNA.chromosome_5.470.1	small subunit ribosomal protein S5	3.82	4.1E-02
mRNA.chromosome_12.413.4	solute carrier family 6	4.37	4.3E-02
mRNA.chromosome_18.1048.1	myosin-3-like	4.82	2.1E-02
mRNA.chromosome_4.1870.1	Brinker DNA-binding domain	4.97	4.1E-02
mRNA.chromosome_15.405.1	folylpolyglutamate synthase, mitochondrial isoform X1	5.15	1.8E-03
mRNA.contig_3725.2.1	RNA-directed DNA polymerase from transposon BS	5.85	2.1E-02
mRNA.chromosome_18.1047.1	myosin-3-like	5.96	1.0E-03
mRNA.chromosome_3.951.1	interferon-induced helicase C domain-containing protein 1	5.98	2.1E-02
mRNA.chromosome_16.1831.1	--	6.30	3.1E-02
mRNA.chromosome_17.2204.1	-- theromacin	9.18	1.4E-02

mRNA.chromosome_17.2202.1	-- theromacin	9.45	2.7E-02

Table S2. DEGs in hemocytes from hemolymph collected from clams in OA vs clams in control conditions. Expression level corresponds to the OA treatment.

Gene ID	Predicted Protein	L2FC	Adj p value
mRNA.chromosome_2.1379.1	protocadherin Fat 1/2/3	-8.20	0.00
mRNA.chromosome_9.119.1	protocadherin Fat 1/2/3	-5.92	0.00
mRNA.chromosome_3.570.1	glutamate receptor ionotropic, NMDA 2A	-5.55	0.04
mRNA.chromosome_19.1763.2	Membrane alanyl dipeptidase (M1) family signature	-5.49	0.05
mRNA.chromosome_11.788.1	glutamate receptor ionotropic, NMDA 2A	-5.23	0.04
mRNA.chromosome_17.1242.1	Integrase catalytic domain profile	-5.00	0.00
mRNA.chromosome_16.1283.1	Integrase catalytic domain profile.	-4.54	0.00
mRNA.chromosome_17.1557.1	Sulfotransferase domain	2.09	0.03
mRNA.chromosome_7.1244.1	Cytochrome P450	2.12	0.01
mRNA.chromosome_2.915.1	neuroligin;	2.19	0.00
mRNA.chromosome_18.134.1	Pol	2.97	0.05
mRNA.chromosome_4.2319.1	cytochrome P450, family 4, subfamily B, polypeptide 1	3.21	0.03
mRNA.chromosome_13.2224.1	prostaglandin-H2 D-isomerase / glutathione transferase	4.54	0.05
mRNA.chromosome_18.1048.1	myosin-3-like	6.11	0.02
mRNA.chromosome_18.1047.1	myosin-3-like	6.57	0.00
mRNA.chromosome_17.2204.1	theromacin	12.98	0.00
mRNA.chromosome_17.2202.1	theromacin	14.17	0.00

Table S3. Excel file. Gene co-expression network analysis of hemocytes from EPF identified six modules of co-expressed genes

Table S4. Excel file. Gene co-expression network analysis of hemocytes from hemolymph identified six modules of co-expressed genes

Table S5. Excel file. Differentially expressed proteins of cell-free EPF from clams in OA vs clams in control conditions. Expression level corresponds to the OA treatment.

Table S6. Excel file. Differentially expressed proteins of plasma (cell-free hemolymph) from clams in OA vs clams in control conditions. Expression level corresponds to the OA treatment.

Table S7. Seawater chemistry ($mean \pm$ SD)

	**Control**	**Acidified**
**pH_T**	8.01 ± 0.24	7.27 ± 0.06
**Temperature (°C)**	25.5 ± 0.53	25.5 ± 0.53
**Salinity PSU**	30 ± 0	29.63 ± 1.06
**pCO_2 ppm**	626.19 ± 190.27	2732.56 ± 338.74
**$\Omega_{aragonite}$**	2.19± 0.24	0.69 ± 0.15
**$\Omega_{calcite}$**	3.23 ± 0.49	0.99 ± 0.15
**DIC**	2061.69 ± 104.40	2313.06 ± 242.12
**CO_3**	133.59± 20.25	38.52± 4.44
**Total Alkalinity**	1988.57± 725.60	2283.97 ± 217.18

Table S8. Primers used for the QPX qPCR assay (produced by Integrated DNA Technologies, Coralville, Iowa)

**Amount**	**Forward**	**Reverse**
2 μM	Forward = 5.8S24For = 5'-TTTAGCGATGGATGTCT-3'	Reverse = QPXITS2-R2 = 5'-GCCCACAAACTGCTCTWT-3'
